# Transcriptomic analyses of cacao flavonoids produced in photobioreactors

**DOI:** 10.1186/s12864-021-07871-0

**Published:** 2021-07-19

**Authors:** Adriana M. Gallego, Luisa F. Rojas, Wilmar G. Valencia, Lucía Atehortúa, Aura I. Urrea, Andrew S. Fister, Mark J. Guiltinan, Siela N. Maximova, Natalia Pabón-Mora

**Affiliations:** 1 Grupo de Biotecnología, Instituto de Biología, Universidad de Antioquia, Medellín, Colombia; 2 Grupo de Biotransformación, Escuela de Microbiología, Universidad de Antioquia, Medellín, Colombia; 3 Centro de Investigación, Desarrollo y Calidad CIDCA, Compañía Nacional de Chocolates S.A.S, Km 2 Vía Belén autopista, Medellín-Bogotá, Colombia; 4 Department of Plant Science, Pennsylvania State University, University Park, PA USA; 5 Huck Institutes of the Life Sciences, Pennsylvania State University, University Park, PA USA; 6 Present address: Pairwise Plants, 110 TW Alexander Dr, Durham, NC USA; 7 Grupo Evo-Devo en Plantas, Instituto de Biología, Universidad de Antioquia, Medellín, Colombia

**Keywords:** Flavonoids, Gene expression, Oxidative stress, Photobioreactors, *Theobroma cacao*

## Abstract

**Background:**

*Theobroma cacao* is a major source of flavonoids such as catechins and their monomers proanthocyanidins (PAs), widely studied for their potential benefits in cardiovascular diseases. Light has been shown to promote plant secondary metabolite production in vitro. In this study, cacao cells cultured in 7.5 L stirred tank photobioreactors (STPs) were exposed to a change of white to blue LED lights for 28 days (d).

**Results:**

Transcriptomic analyses were performed in three time points comparing changing expression patterns, after cell exposure to white light (d0-VS-d14), after a shift from white to blue light (d14-VS-d15), and after an extended period of blue light for the following 15 days (d15-VS-d28). Under white light, there was enrichment in metabolic pathways associated with cell growth (carbon, glycolysis, and amino acid biosynthesis) accompanied by a significant increase in the PAs content. In the shift to blue light, further increase in PAs content was observed concomitantly with the significant expression of *TWO-COMPONENT RESPONSE REGULATOR* genes involved in the early stress responses via circadian clock and hormone pathways. Under blue light exposure, we observed a depletion of PAs content associated with ROS-mediated stress pathways.

**Conclusions:**

Light effects on large-scale cell cultures in photobioreactors are complex and pleiotropic; however, we have been able to identify key regulatory players upstream cacao flavonoid biosynthesis in STPs, including *TWO-COMPONENT SYSTEM* and ROS-signaling genes. The crosstalk between flavonoid biosynthesis and regulatory networks led to understand the dynamics of flavonoid production and degradation in response to light-driven ROS signals. This can be used to optimize the time, and the yield of in vitro targeted metabolites in large-scale culture systems.

**Supplementary Information:**

The online version contains supplementary material available at 10.1186/s12864-021-07871-0.

## Background

Plant polyphenols are secondary metabolites used as pharmaceuticals, food additives, and flavors, among others [1]. In particular, *Theobroma cacao*, the chocolate tree, is an exceptional source of dietary polyphenols [2]. Cacao polyphenols comprise mainly flavonoid-subgroups catechins (29–38%), anthocyanins (4%), and proanthocyanidins (58–65%) [3]. The regular intake of cocoa polyphenols in the diet reduces the risk of cardiovascular disease through their antioxidant properties [4]. Furthermore, cacao polyphenols can reduce blood pressure and improve cognitive performance [5].

Polyphenol and downstream synthesis pathways are generally stimulated in response to biotic or abiotic stresses such as pathogen attacks, UV-irradiation, wounding, nutrient deficiencies, extreme temperatures, herbicide treatments and light [6]. Light is one of the most important environmental factors that regulate polyphenol production and accumulation, but a comprehensive model connecting photoreceptors to the light-driven downstream genes is still lacking [7–9]. It is known that plant exposure to short-wavelengths like UV-B and blue light mediate defense signaling pathways that lead to the synthesis of different secondary metabolites including carotenoids, anthocyanins, aliphatic glucosinolates, and epicatechin as a result of stress [7, 10–13]. However, high light levels or long-light exposure can lead to metabolite degradation [14]. These metabolites contribute to long-term adaptation to biotic and abiotic stressors [15].

Light regulates plant gene expression via a network of interconnected receptors and signal transduction pathways that integrate light quantity and quality to drive the regulation of thousands of genes. In the light signaling cascade, key transcription factors have been identified, including *CONSTITUTIVE PHOTOMORPHOGENESIS 1* (*COP1*), which activates *LONG HYPOCOTYL 5* (*HY5*). The *HY5* transcription factor responds positively to blue light through a G-box motif and controls downstream transcription of MYB genes, which in a combinatory fashion directly control the flavonoid pathway [9, 16, 17]. Furthermore, a direct light regulation over flavonoid structural genes has been associated with the presence of light regulatory units (LRUs) in promoters. The LRUs, consisting of a MYB-recognition element (MRE) and an ACGT-containing element (ACE) have been found in the promoters of *CHALCONE SYNTHASE* (*CHS*)*, CHALCONE ISOMERASE* (*CHI*)*, FLAVANONE 3 HYDROXYLASE* (*F3H*) and *FLAVANOL SYNTHASE* (*FLS*) in Arabidopsis and their presence was correlated with flavonoids activation to light inputs [18].

In addition, UV and blue lights trigger the synthesis of reactive oxygen species (ROS), resulting in the activation of signaling and defense responses driving flavonoid production [19, 20]. The ROS production in both plants and animals is part of the normal metabolism of mitochondria and peroxisomes. Additionally, ROS are key signaling molecules in plant adaptation and stress response [21]. However, an increase of ROS under stress conditions could result in DNA damage and protein and lipid oxidation [22]. ROS molecules in cells are mainly represented by superoxide anion (O_2_^−^), singlet oxygen (^1^O_2_), and hydrogen peroxide (H_2_O_2_) [23]. It is known that ROS accumulate in the nucleus after cryptochrome activation under blue light [24], but in general, the knowledge of the relationship between light and ROS in plants at molecular level is limited.

Additionally, genes implicated in pathogen defense, biotic and abiotic stresses are more highly transcribed due to ROS-induction mediated by cryptochromes [25]. The repeatability of transcription profiles in response to ROS has resulted in marker genes that indirectly detect ROS accumulation [26]. However, the expression analyses of ROS gene markers under different light conditions in cell cultures at bioreactor scale and specifically their effect in cacao flavonoid production has not yet been studied.

Plant cell cultures have facilitated the study of the light effects on the production of phenolic compounds [9, 27, 28]. However, so far, most experiments of light-responsive cacao cell cultures have been done at small scales, limiting the industrial scale of flavonoids mass production. Bioreactors represent an ideal large scale automated system with major improvements over flask systems due to higher volumes and the precise regulation of physicochemical factors like gaseous composition, efficient oxygen transfer, pH and hydrodynamic forces [29]. Also, bioreactors can guarantee large-scale commercial production of secondary metabolites, including phenolic compounds with low production costs [30–33].

We have recently reported that light has a significant effect on structural and regulatory gene expression associated with the flavonoid pathway in cacao cells cultured in flasks [9]. Specifically, a white light followed by a blue light exposure can trigger metabolic changes inducing a faster accumulation of phenolic compounds and shifting flavonoid profiles in terms of the epicatechin/catechin ratios [9]. In the present study, we investigated changes in gene expression related to flavonoid production in cacao cell cultures cultured  in 7.5 L STPs, under white and blue light treatments. Particularly, we focused on expression changes of photoreceptors and light-responsive downstream genes, light-induced ROS markers and flavonoid structural genes during 28 days of the experiment. Finally, we present a hypothetical model of the light-induced transcriptional regulation of the dynamic between cacao flavonoid production and degradation at a bioreactor scale.

## Results

### Effect of the light treatments in the total proanthocyanidins content (PAs) at bioreactor scale

**Fig. 1:**
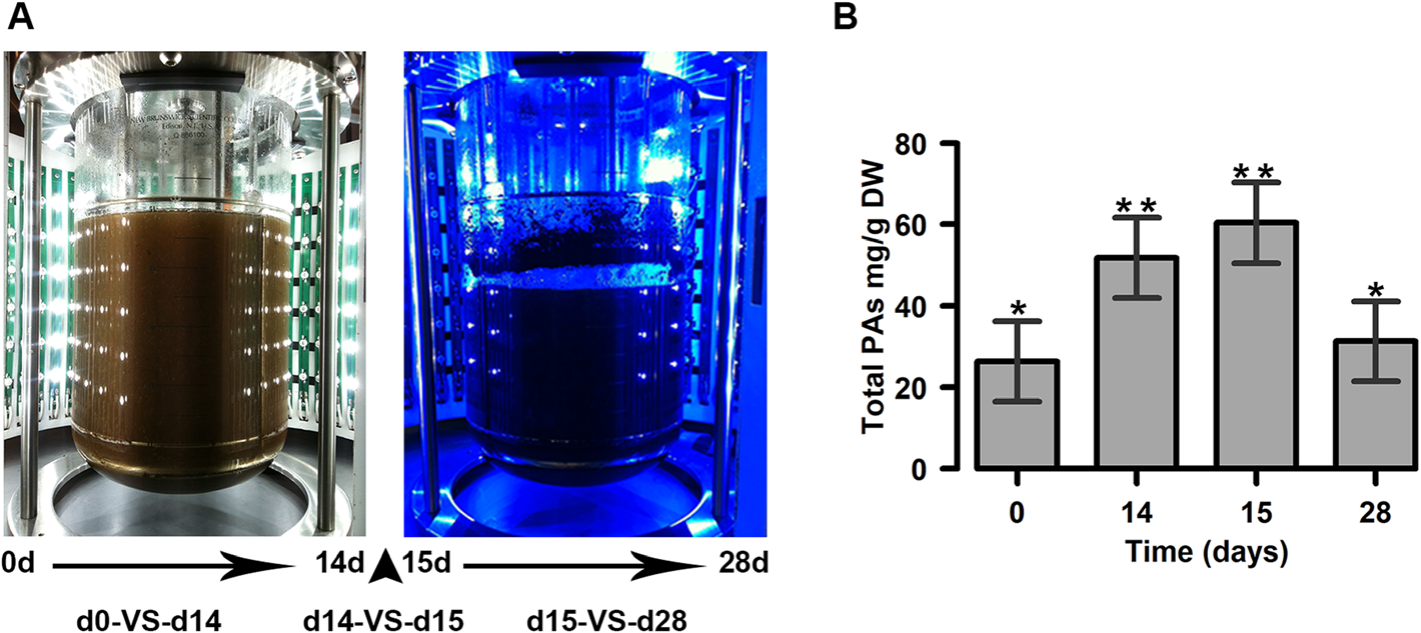
Experimental design and PAs  accumulation in cacao cell cultures grown in stirred tank photobioreactors (STPs). **a** Bioreactors under white (Day 0-14) and blue (Day 15-28) light conditions. Fresh media was added at day 14 (arrowhead). Three comparisons were performed: d0-VS-d14, d14-VS-d15, and d15-VS-d28. **b** Total proanthocyanidins (PAs) content measured in cacao cell cultures grown in STPs. Treatments showing a different number of asterisks are considered significant with *p*-value < 0.05. Bars represent probability intervals. DW: dry weight

To investigate the light impact on flavonoid accumulation in cacao cells at STPs, we followed an experimental design similar to priviously reported small -scale flask system that resulted  in an increase in PA content accompanied with changes in the flavonoid profiles [9]. The light change from white to blue took place once between 14 and 15 days, with no changes in light intensity. Although we aimed to specifically test the impact of light on STPs metabolism, this large-scale setting may have added effects of light and culture time (i.e., the age of the culture). The independent effects that the two variables may have will require future large-scale analyses to be conducted in parallel under each light conditions for 28d. Nevertheless, our experiment does provide a general overview of STPs metabolic changes when compared to small-scale cultures. The total proanthocyanidins (PAs) content was measured in four time-points at days 0, 14, 15 and 28 with three replicates for each treatment and taken from three independent bioreactor experiments. At the end, all data were pooled for each time point (Fig. 1a). Compared to day 0 (26.37 mg/g), a statistically significant increase of PAs production (*p*-value < 0.05) was observed at day 14 (51.84 mg/g). A slight increase in the PAs was recorded during the transition from white to blue light (60.41 mg/g at 15), which was not statistically significant. Finally, during long blue exposure, from 15 to 28 days, the PAs content decreased to 31.28 mg/g (Fig. 1b).

### Transcriptome sequencing and global annotation

High-throughput RNA-Seq of cells in STPs generated 16.49–23.39 million (M) 100-bp single-end reads per sample. After the quality filtering process, 99.09% of the reads remained, with a Q36  ≥ 85%. The clean read count per library ranged from 16.29 to 23.31 million. The percentages of mapped reads generally ranged between 80.12–95.58% (Table S1). Reads mapped to approximately 18,800 genes which are about 75% of the 24,831 genes present in the V2 Criollo cacao genome database (http://cocoa-genome-hub.southgreen.fr/). In total, 19,263 non-redundant genes were identified, corresponding to 77.57% of cacao genes. GO terms showed that most genes were grouped in the cell part and cell (96%) for the cellular component. For the biological process component, the categories of cellular process (67%), metabolic process (56%) and response to the stimulus (46%) were enriched. Finally, the binding category (55%) was enriched in the molecular function (Fig. S1). COG analyses identified 1347 genes in function unknown (S), 702 in general function prediction (R), 270 in amino acid transport and metabolism (E) and 88 genes in secondary metabolites biosynthesis, transport and catabolism (Q) (Fig. S2).

### Data exploration and pathway analysis

To detect differentially expressed genes (DEGs) in the cacao cells, three pairwise comparisons were made among the following sampling points at d0-VS-d14 under white light, at d14-VS-d15 reflecting the difference at 24 h after change from white to blue light and at d15-VS-d28 under long blue light exposure. In the three pairwise comparisons, we identified 4908 (d0-VS-d14), 54 (d14-VS-d15) and 536 (d15-VS-d28) non-redundant DEGs for a total of 5248. From these, only four were shared between the three pairwise comparisons, while 19 DEGs were shared between d0-VS-d14 and d14-VS-d15; eight DEGs were shared between d14-VS-d15 and d15-VS-d28 and finally, 215 were shared between d0-VS-d14 and d15-VS-d28 (Fig. S3).

**Fig. 2:**
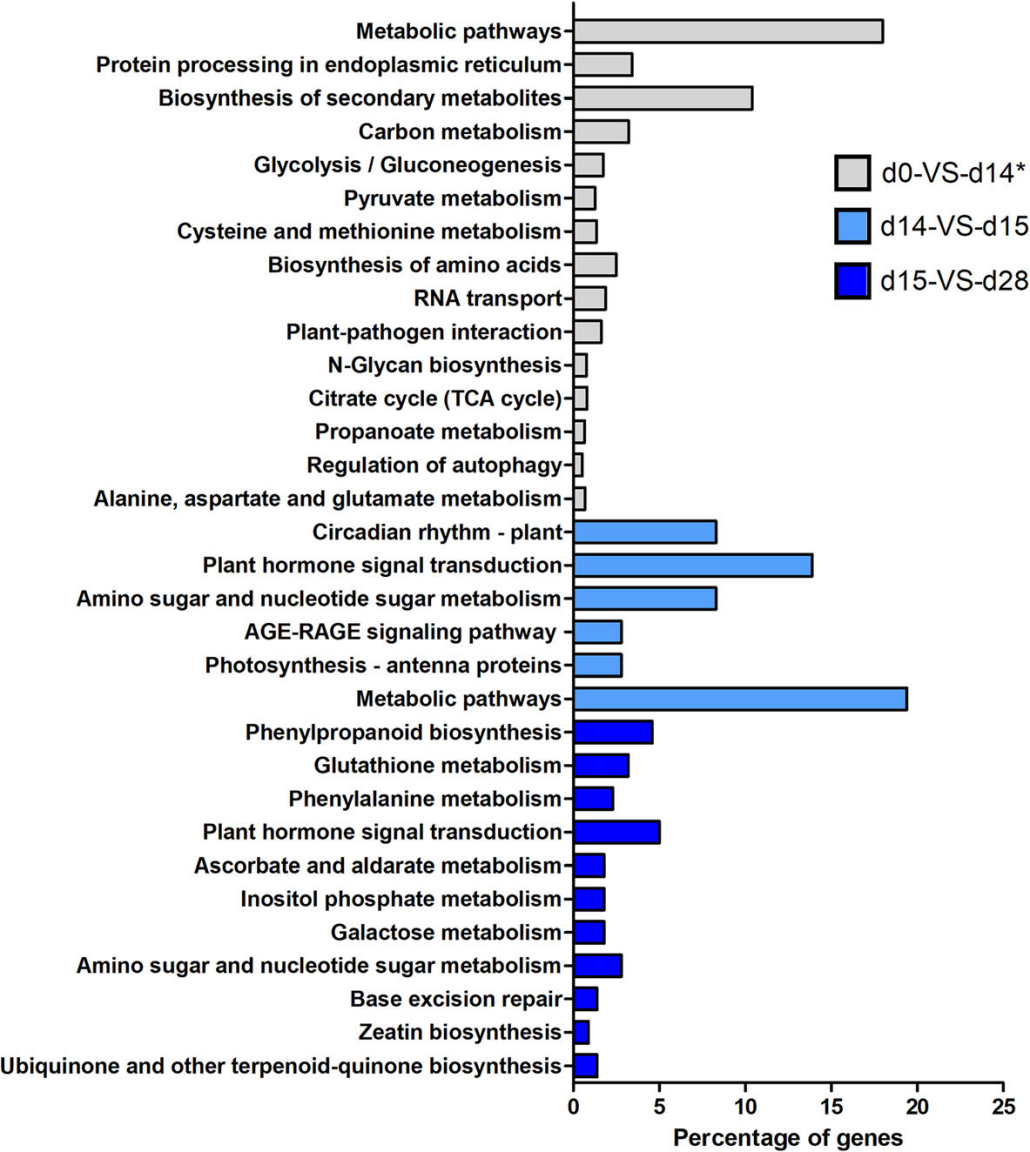
Enrichment analysis of DEGs in three pairwise comparisons for cacao cells grown in STPs under the light treatments shown in Fig. 1. Categories presented were significantly enriched between the comparisons in the conventions (*p*-value < 0.05). *Only the top 15 of the significant DEGs are shown for d0-VS-d14

A KEGG metabolic enrichment analysis was performed on the DEGs to detect the more abundant categories during the experiment (Fig. 2, Table S2). At d0-VS-d14, metabolic pathways and biosynthesis of secondary metabolites were significantly abundant. Interestingly, 14 flavonoid genes were enriched in these two categories. At d14-VS-d15, categories like circadian rhythm, plant hormone signal transduction, photosynthesis, sugar metabolism, and metabolic pathways were significantly enriched. Finally, at day d15-VS-d28, phenylpropanoid biosynthesis, ascorbate and glutathione metabolisms, plant hormone signal transduction and sugar metabolism were detected as significantly enriched categories. Interestingly, the category for metabolic pathways was shared between the three pairwise comparisons, while plant hormone signal transduction and aminoacid/sugar metabolism categories were shared exclusively between comparisons of blue light treatments (d14-VS-d15 and d15-VS-d28).

Next, we clustered DEGs based on their expression patterns throughout the experiment and performed a KEGG analysis to assess the light impact on global gene expression (Fig. S4). The analysis revealed nine significant clusters (*p*-value < 0.05) with different expression patterns varying according to the light treatments (Fig. S5). Clusters 46 and 48, enriched in categories like metabolic pathways, biosynthesis of secondary metabolites and flavonoid biosynthesis, showed positive regulation in both light treatments, with higher expression under blue light. Cluster 49 and 45, enriched in carbon metabolism, RNA transport, and the glycolysis and gluconeogenesis pathways showed a positive regulation under white light while lacking expression under blue light. Clusters 39 and 47, enriched in protein processing in the endoplasmic reticulum, ribosome biogenesis, and glycine metabolism, showed a positive regulation until day 15 (d0-VS-d14 and d14-VS-d15) but negative regulation afterward (d15-VS-d28). Clusters 2 and 13, enriched in metabolic pathways, the pentose phosphate pathway, protein processing in the endoplasmic reticulum, phenylpropanoid biosynthesis and biosynthesis of secondary metabolites showed negative regulation under white light but positive regulation under blue light. Finally, cluster 12, which included plant-pathogen interaction, endocytosis and plant hormone signal transduction, showed a negative correlation to white light and remained unchanged under blue light.

The comparison of DEGs during the time course of the experiment resulted in 359 transcription factors (TFs) identified. A total of 322, 5 and 32 TFs were detected for d0-VS-d14, d14-VS-d15 and d15-VS-d28 respectively. Only one TF (Tc04v2_t015040) was common for all three pairwise comparisons, corresponding to NAC member [no apical meristem (NAM), Arabidopsis transcription activation factor (ATAF1/2) and cup-shaped cotyledon (CUC)]. For d0-VS-d14, the most abundant families belonged to ERF, MYB, NAC, WRKY, and bHLH. For d14-VS-d15 TF members of the NAC, ERF, LBD, and HSF families were found. For d15-VS-d28, the most abundant TF families were ERF, MYB, NAC, WRKY, and bHLH (Table S3).

### Photoreceptors and light signal perception

**Fig. 3:**
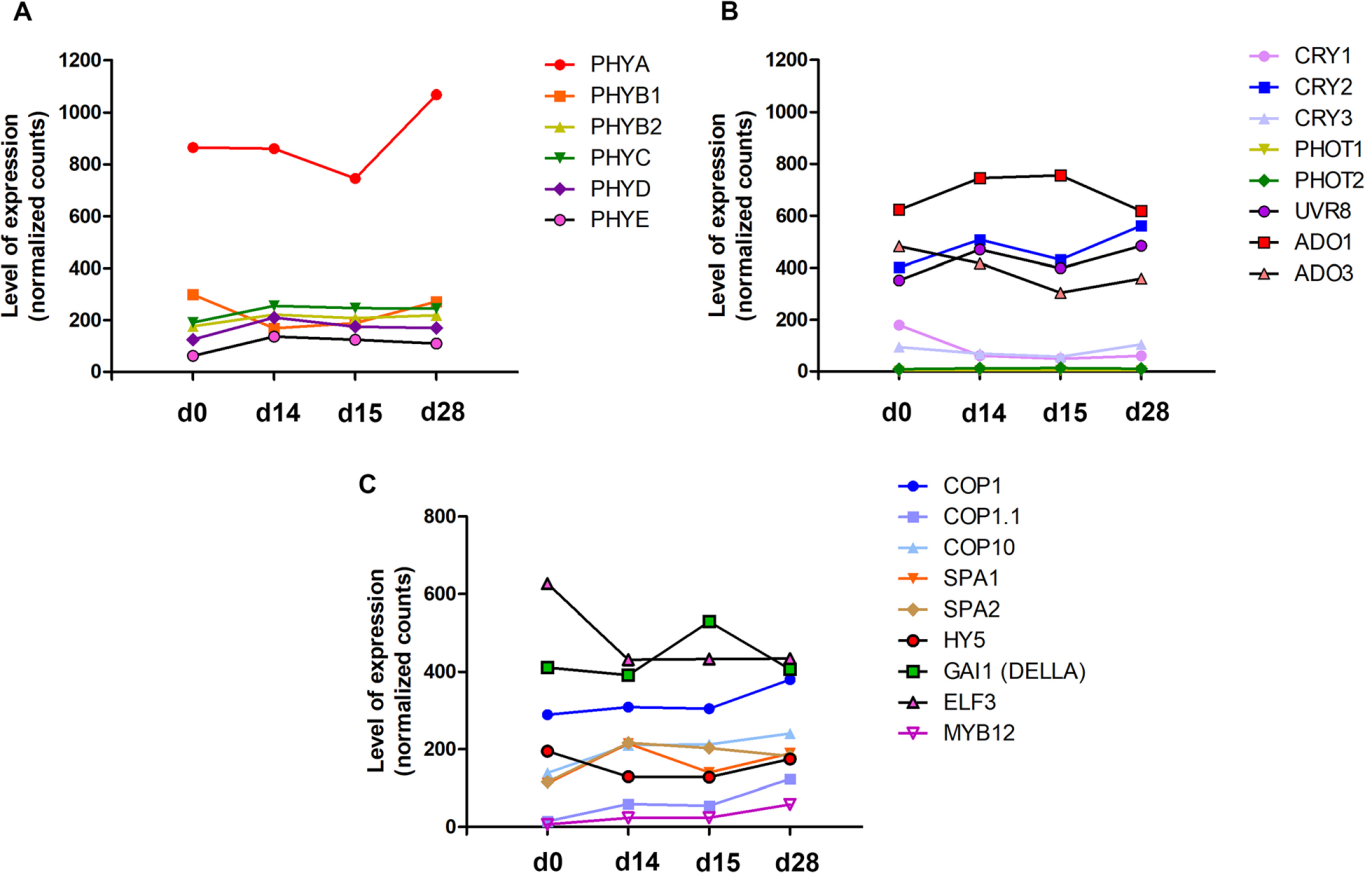
Gene expression patterns of light-signaling genes identified in cacao cells grown in STPs. **a** Red/Far-red responsive photoreceptors. **b** Blue light and UV responsive photoreceptors. **c** Downstream light signaling genes. The data corresponds to the mean of three biological replicates. d, day. Note that *PHYE* and *COP1.1* were found to be differentially expressed under d0-VS-d14 and d15-VS-d28 respectively

To identify which photoreceptors and their target genes showed high gene expression levels under the light treatments, we performed a detailed expression analysis of the selected red/far-red and blue photoreceptors (Fig. 3a-b) and downstream light genes like *COP1*, *COP10*, *SPA1*, *SPA2*, *HY5*, *GAI1*, *ELF3* and *MYB12* (Fig. 3c) identified in the cacao global transcriptome. From the red/far-red photoreceptors, 4 out of 6 showed upregulation under white light (d0-VS-d14) and a slight reduction under blue light treatments (d14-VS-15 and d15-VS-d28). *PHYB1* showed an opposite trend of downregulation  under white light and upregulation under blue light. On the other hand, *PHYA* showed a higher expression level compared to the other photoreceptors after the long blue exposure (d15-VS-d28). The blue-light photoreceptors, *ADO3*, *CRY1,* and *CRY3* showed similar expression patterns being downregulated at d0-VS-d14 and d15-VS-d28 and upregulated at d15-VS-d28. *CRY2* and *UVR8* showed similar expression trends, being upregulated at d0-VS-d14 and further increasing their expression at d15-VS-d28. Remarkably, they showed a slight downregulation in the shift from white to blue light (d14-VS-15). *ADO1* showed the highest levels of expression, followed by *CRY2* and *UVR8* among all blue receptors. *ADO1* was upregulated at d0-VS-d14 and d14-VS-15 and decreased at d15-VS-d28. *PHOT2* showed the lowest and the most invariable expression throughout the time course. The annotation of photoreceptors and statistical information between treatments are described in Table S4.

Downstream light signaling genes were also studied in detail, as they are the first responders in the photoreceptors cascade. First, the two transcripts of *COP1* (*COP1* and *COP1.1*) showed upregulation at d0-VS-d14 and d15-VS-d28 but not for d14-VS-d15. *COP10* and *MYB12* showed a positive regulation throughout the time course of the experiment. In contrast, *SPA1* and *SPA2* showed a positive activation at d0-VS-d14, then downregulation at d14-VS-d15 and showed opposite expression patterns at d15-VS-d28. The transcription factor *HY5* was only upregulated under long blue light exposure. Finally*, GAI1* and *ELF3* were downregulated at d0-VS-d14 but upregulated under blue light in d14-VS-15 and showed opposite expression patterns at d15-VS-d28.

### ROS markers and light signal perception

**Fig. 4:**
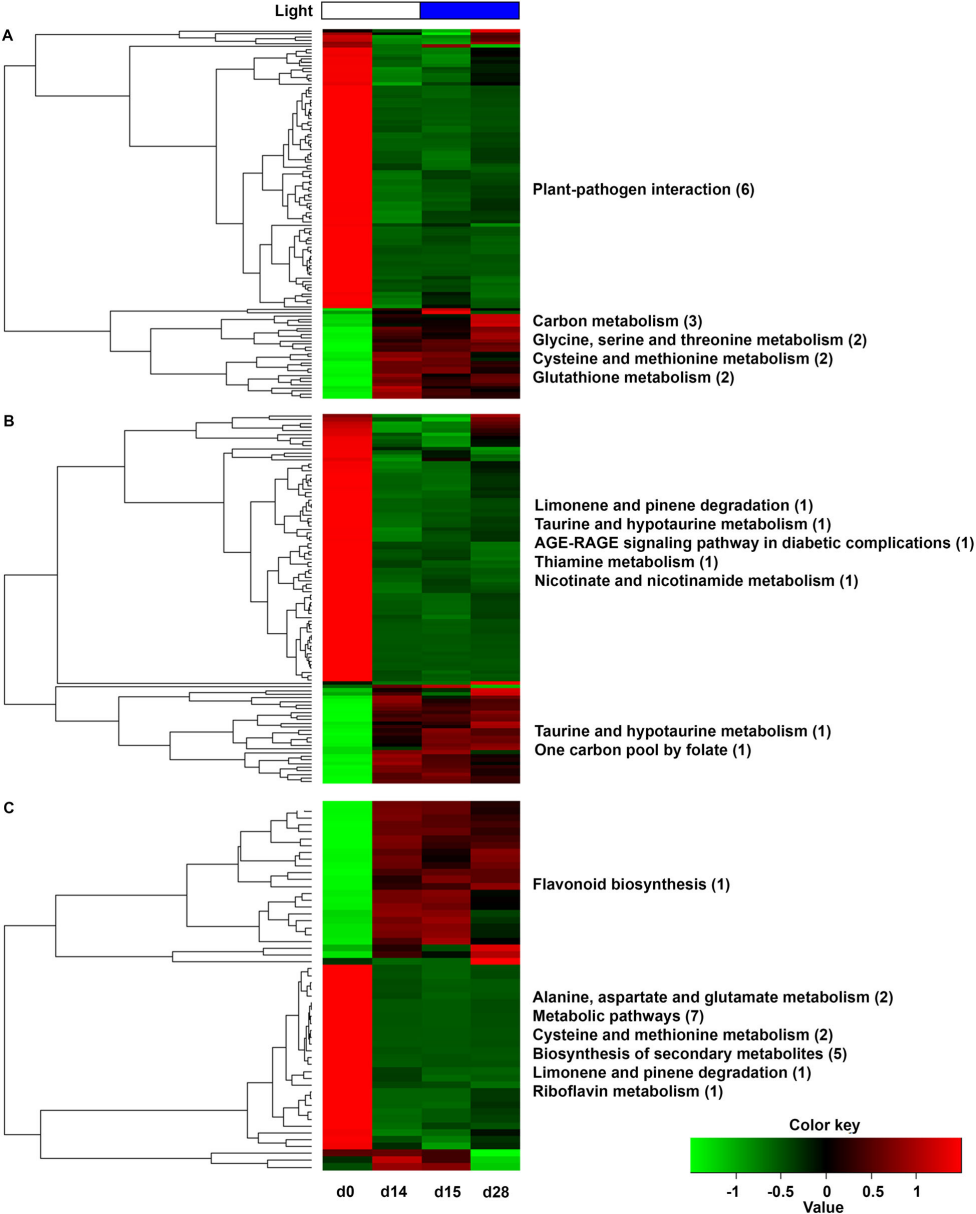
Cluster analysis showing the dynamics of ROS markers gene expression under white-blue treatments of cacao cells grown in STPs. DEG genes were subjected to metabolic pathway KEGG enrichment analysis for each type of ROS. Only significant enriched categories are shown (*p*-value < 0.05). Upregulated and downregulated genes are colored in red and green respectively. **a** Singlet oxygen (^1^O_2_). **b** Hydrogen peroxide (H_2_O_2_). **c** Superoxide (O_2_^−^). d, day

To identify candidate genes with potential roles in ROS signaling under light conditions, the 5248 cacao DEGs were compared to 832 abiotic stress ROS gene markers previously reported for Arabidopsis [26]. These reported abiotic stress markers represented three types of ROS. The resulting analysis allowed us to retrieve 118, 101 and 54 candidate markers for singlet oxygen, hydrogen peroxide and superoxide respectively (Fig. 4). Interestingly, we noticed that most of the DEGs for singlet oxygen and hydrogen peroxide were downregulated throughout the time course of the experiment. Conversely, superoxide ROS markers had equal rates of up and downregulation during the experiment.

We identified and annotated the ROS markers up and downregulated (up/down) for the three pairwise comparisons. For the singlet oxygen category, 28/86 (up/down), 0/1 (up/down) and 14/11 (up/down) were differentially expressed at d0-VS-d14, d14-VS-d15 and d15-VS-d28 respectively. Cluster analysis showed plant-pathogen interaction enriched in the downregulated genes, instead, carbon metabolism, amino acids, and glutathione metabolisms were enriched for upregulated genes (Fig. 4a). For hydrogen peroxide, 24/68 (up/down), 0/0 (up/down) and 14/2 (up/down) genes were differentially regulated in the same three pairwise comparisons. Limonene and pinene degradation, taurine and hypotaurine, thiamine and nicotinate metabolisms were categories enriched in downregulated genes instead taurine and one carbon pool by folate were in the upregulated DEGs (Fig. 4b). Finally, in the case of superoxide, 23/27 (up/down), 0/1 (up/down) and 4/3 (up/down) DEGs were identified respectively in the three pairwise comparisons (Fig. 4c). Interestingly, flavonoid biosynthesis was the only  enriched significant category in the upregulated DEGs. On the other hand, alanine, aspartate, and glutamate metabolism, biosynthesis of secondary metabolism and riboflavin metabolism were the significantly enriched categories in the downregulated DEGs (Table S5).

We detected some interesting candidate genes previously reported in the literature to be involved in oxidative stress. For the singlet oxygen, transcripts were annotated as copper transport proteins, glutathione S transferase, cysteine-rich receptor kinase, ethylene-responsive and MYB transcription factors under white light and glutathione S transferase and ATPase under blue light. For hydrogen peroxide, we detected *NAC29*, *SERINE HYDROXY-METHYLTRANSFERASE*, *ASCORBATE PEROXIDASE*, *STRESS PROTEIN*, *MITOGEN-ACTIVATED PROTEIN KINASE*, *HEAT STRESS PROTEINS* and *ONE COPPER TRANSPORTER* under white light and *MYB108* and a putative *TIFY* under blue light. For superoxide, the flavonoid gene *ANS*, a *GATA* transcription factor, a *PEPTIDYL-PROLYL TRANS ISOMERASE*, and a *CYTOCHROME P450* were detected under white light, and instead, *GIGANTEA*, hormonal signaling (small auxin up RNAs, SAURs) and others uncharacterized genes were upregulated under blue light conditions (Table S6).

Furthermore, we identified a total of 20 TFs as a subcategory among all cacao ROS markers. Out of the group of DEGs linked to single oxygen ROS category, eight TFs differentially expressed were annotated as members of the ERF, bHLH, MYB, WRKY and TALE families. In the hydrogen peroxide analysis, another set of eight TFs was annotated as NAC, MYB, bHLH, ERF and HSF families. Finally, for superoxide, we identified four TFs that were representative members of the MYB-related, HB, bZIP and Dof families (Table S7). Additionally, we analyzed the levels of expression for three antioxidant enzymes, superoxide dismutase (SOD), catalase (CAT) and ascorbate peroxidase (APX) thoughout the light treatments (Fig. S6). The results presented in this study, demonstrate that light conditions lead to oxidative stress in the cells of *T. cacao* and the activity of the enzymes involved in the detoxification processes varies during the kinetics. *SOD* activity increases during the first 14 days of the kinetics and declines afterward. Conversely, *APX* was downregulated during the first 14 days and increases in the shift to long blue light and during the remaining light exposure. Finally, *CAT* decreased during the first 14 days and no significant change was evident during blue light exposure.

### Identification and expression analysis of flavonoid biosynthetic genes under light treatments

**Fig. 5:**
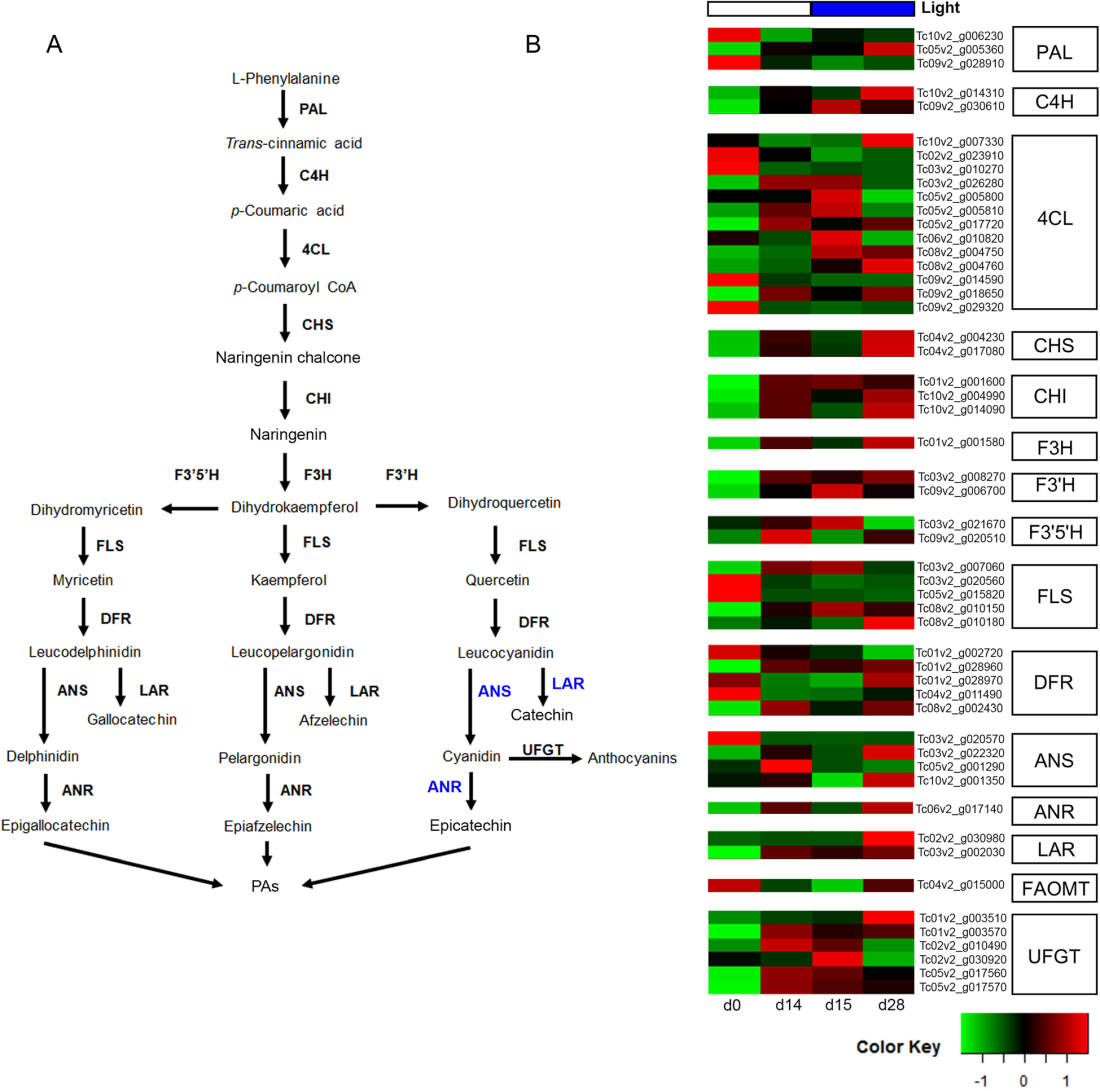
Flavonoid gene expression pattern of cacao cells grown in STPs. **a** Flavonoid biosynthetic pathway, late structural genes are in blue. **b** Flavonoid biosynthethic gene expression changes in CCBs. d, day; PAL, phenylalanine ammonia lyase; C4H, cinnamate 4-hydroxylase; 4CL, 4-coumarate coenzyme A ligase; CHS, chalcone synthase; CHI, chalcone isomerase; F3H, flavanone 3-hydroxylase; F3’H, flavanone 3′-hydroxylase; F3’5’H, Flavonoid-3′,5′-hydroxylase; FLS, Flavonol synthase; DFR, dihydroflavonol-4-reductase; ANS, anthocyanidin synthase; ANR, anthocyanidin reductase; LAR, leucoanthocyanidin reductase; FAOMT, Flavonoid 3′,5′-methyltransferase; UFGT, UDP-Glucose: Flavonoid 3-O- Glucosyltransferase. d, day

We identified 52 putative transcripts associated with all flavonoid pathway genes (Fig. 5a). Fifty-two structural gene homologs were found from *PHENYLALANINE AMMONIA-LYASE* (*PAL*) up to *LEUCOANTHOCYANIDIN REDUCTASES* (*LAR*). Also, seven homologs were identified for modifier proteins involved in metilation and glucosylation of flavonoids, one *FLAVONOID 3′,5′-METHYLTRANSFERASE* (*FAOMT*) and six *UDP-GLUCOSE: FLAVONOID 3-O- GLUCOSYLTRANSFERASE* (*UFGT*) (Fig. 5b).

Analyzing the levels of expression, we identified  that most of the cacao flavonoid biosynthetic genes showed a trend. An upregulation at d0-VS-d14 (white light) followed by downregulation at d14-VS-d15 (shift white to blue) and again upregulation at d15-VS-d28 (long blue light exposure). An excepion was observed for a copy of *ANS* (Tc03v2_g020570), which was downregulated by the white light at d0-VS-d14 and remained largely unaffected by the blue light (d14-VS-d15 and d15-VS-d28) (Fig. S7). Interestingly we detected that *CHS* (Tc04v2_g017080) and *ANS* (Tc03v2_g022320) had the highest expression levels overall flavonoid structural genes throughout the experiment. These genes were followed by intermediate levels of expression of *CHI*, *CHS*, *ANR* and *LAR* copies. Finally, *DFR* and *F3’5’H* showed the lowest levels during the time-course experiment.

### Light regulatory unit analysis in the promoters of flavonoid genes

**Fig. 6:**
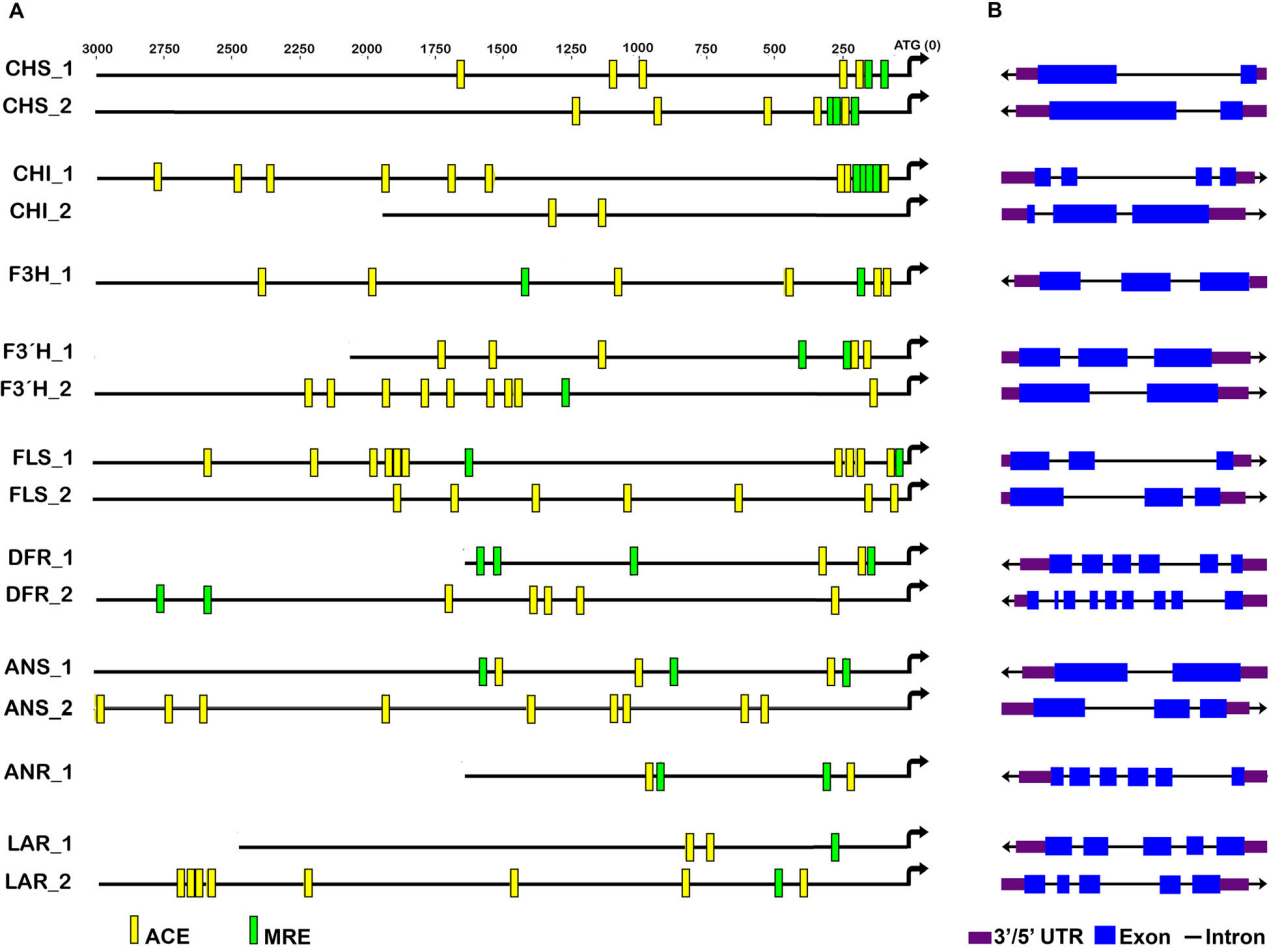
In silico Light Regulatory Unit (LRU) analysis on the flavonoid promoters in cacao cells grown in STPs. **a** Positions of the MYB-recognition element (MRE, green bars) and an ACGT-containing element (ACE, yellow bars) which conform a light regulatory unit in the cacao flavonoid structural gene promoters. Gene names in the left. Positions are relative to the transcription start site for each gene (black arrow). **b** Gene map for each cacao flavonoid gene

To track putative light response of the biosynthetic flavonoid pathway genes, we searched for light regulatory units (LRUs) consisting of MYB recognition elements (MRE) and ACE sequences in the promoter regions of structural genes. The in silico analysis revealed that all structural genes include at least one LRU (Fig. 6). The consensus sequences for ACE and MRE were cACGTg and CTACC, respectively. Promoters of early biosynthetic genes showed higher ratios of ACE/MRE compared to the remaining structural genes, particularly both copies of *FLS*. Interestingly, individual copies of *ANS* and *ANR* showed equal numbers of MRE and ACE elements. Also, both copies of *LAR* presented a unique MRE element (Fig. 6a). Furthermore, gene copies do not exhibit similar LRUs, suggesting that paralogs can be regulated differently. Finally, gene maps evidence that a higher number of exons are present for late biosynthetic genes (*DFR, LAR, ANR*) compared to early genes, except for *ANS* (Fig. 6b).

### qRT-PCR validation of differentially expressed genes

To further validate the RNA-seq results, we randomly selected 6 DEGs, five genes associated with the flavonoid pathway including *CHS, DFR, ANS, ANR, LAR* and one light-signaling regulatory *NAC* gene for qRT-PCR analysis and fold changes at 0d-VS-14, 14d-VS-15d and 15d-VS-28d (Table S8). The qRT-PCR results were highly correlated  with the RNA-seq data with correlation coefficient 0.94 (Fig. S8).

## Discussion

### Light-driven gene expression in cells at STPs producing flavonoids

Different light conditions have been reported to induce secondary metabolites in vitro in several plant species [34, 35]. In cacao cells cultured in flasks, flavonoid biosynthesis has been described to occur under white and blue lights as well as in the dark [9, 36]. However, the effects of long-term exposure and shifts between different light conditions have not been evaluated in large-scale culture systems for cacao. Here we present the first comprehensive expression analysis of cacao cells growing in  STPs under white followed by blue light. We describe the most prevalent cellular processes occurring under three different light regimes. The white light treatment (d0-VS-d14) was characterized by a significant increase of PAs content and the enrichment in metabolic pathways associated with cell growth (carbon, sugar, aminoacids). After the shift to blue light (d14-VS-d15) there was a further increase in PAs content concomitantly with the enrichment in early stress responses of the circadian clock and plant hormone signaling. Finally, long blue light exposure (d15-VS-d28) resuted in PA depletion  and it was linked to ROS-mediated stress pathways.

Photoreceptors are pivotal in the light-signal transduction mechanisms, including specific protein-protein interactions, downstream enzyme activities and developmental responses [37]. Two alternative pathways have been proposed downstream photoactivation that induce plant developmental processes, the COP1 mechanism and the Phytochrome-Interacting Factor (PIF) pathway [38–40]. In the cacao cells growing in  STPs, the photoreceptors and some downstream light signaling genes like *COP1*, *SPAs*, *HY5* and *MYB12* were differentially expressed in white vs blue light. In general, the highest levels of expression were recorded for *PHYA*, *ADO1*, *CRY2*, *GAI1* and *COP1*, suggesting a key role of these genes in perceiving the light inputs. However, in comparison to *COP1*, the *PIFs* showed deficient expression in response to light, indicating that photo-morphogenesis processes mediated by *PIFs*, are not likely occurring in the cells at STPs. Thus, we hypothesized that two consecutive processes occurred  at bioreactor scale: (1) *COP1* pathway is preferentially governing light downstream signaling and (2) both photoreceptors and downstream genes are interconnected to promote the detected PA biosynthesis.

Additionally, we identified major metabolic processes occurring under each light treatment that explain differences in the PA content for the time-points sampled. Under white light (d0-VS-d14), the PA increase was correlated with metabolic pathways associated with cellular growth, including carbon, glycolysis, protein processing and biosynthesis of amino acid metabolisms. For this study, we focused on the metabolic pathways co-expressing with flavonoid biosynthesis that could be correlated to the increase in PA content observed. Particularly the porphyrin/chlorophyll metabolism co-expressed with flavonoids and it was significantly enriched in white light. In the porphyrin/chlorophyll category, *GLUTAMATE-TRNA LIGASE* (*GluRS*), *MAGNESIUM-CHELATASE SUBUNIT CHLI* (*GUN4*) and *DIVINYL CHLOROPHYLLIDE A 8-VINYL-REDUCTASE* (*DVR*) genes showed upregulation under white light. These genes localize in the chloroplast and are involved in the biosynthesis of chlorophyll and heme molecules, essential for light-harvesting and energy transduction in photosynthesis and the cell growth [41–44]. The flavonoids are thought to participate in protecting the photosynthetic apparatus against photoinhibition under excessive light [45]. However, two other functions have been assigned to the flavonoids in plants exposed to high light. On one hand they can act like carbon sinks that absorb excess photosynthetic carbon (i.e., when the synthesized carbon from photosynthesis surpasses growth needs, it is used for the synthesis of secondary metabolites); on the other hand, flavonoids can perform antioxidant functions in leaves exposed to high sunlight [46]. The upregulation of both chlorophyll biosynthesis and 14 flavonoid biosynthetic genes occurred in the first 14 days of white light exposure during an active growth stage of the cacao cells. The expression of some of these flavonoid genes was validated by qRT-PCR. Thus we lean towards the hypothesis that flavonoids function at d0-VS-d14 could be likely associated with the suitable functioning of PSII [47], as PAs could not exert photoprotection activities over an incipient photosynthetic apparatus.

Interestingly, the concentration of PAs in the cacao cells doubled after 14 days indicating that it took some time for flavonoid synthesis to reach sufficient levels to exhibit any protective effect in cultures exposed to high light. It is possible that during the acclimatization to white light, the defense mechanism of the cells in STPs relayed on the activation of the antioxidant enzymes like SOD. Indeed, the strong and exclusive upregulation of *SOD* in the transcriptome under white light suggested the catalysis of singlet oxygen, a ROS signal promoting the flavonoid biosynthesis [45]. Altogether, the data under white light suggests that PAs production was stimulated by light-driven ROS signals concomitantly with an enrichment in light-harvesting metabolic pathways, where antioxidant activities were supported mainly by the enzymatic defense system addressed by *SOD,* followed by flavonoids participating in a less extension due PAs were being synthetized.

In the shift to blue light exposure (d14-VS-d15), a slight increase of PA content was evidenced concomitantly with the enrichment of the circadian rhythm and plant hormone signaling pathways. These two metabolic pathways were enriched in *TWO-COMPONENT RESPONSE REGULATOR* genes like *ARR7, ARR4*, *ARR9* and *ORR9*. In plants, *ARRs* genes play roles in diverse biological processes, including responses to environmental stress stimuli [48]. For example, light-induced flavonoid accumulation mediated by two-component systems was also reported in *Epimedium pseudowushanense* [49]. It has been established that *ARR4* regulates circadian period and red/far-red light response by its direct interaction with *PHYB* [50, 51]. Interestingly, in our study *ARR4* was exclusively upregulated under blue light, suggesting a blue light-responsiveness of *ARR4* in cacao.

Furthermore, the upregulation of the blue light receptor *ADAGIO1*/*ZEITLUPE1,* (*ADO1/ZTL1*, Tc09v2_t014980) and the clock component *GIGANTEA* (*GI*) suggest that Zeitlupe family members, as well as Zeitlupe-responsive genes like *GI*, can have a more prominent role perceiving early blue light inputs in cacao cells. The relationship between *ZTL* and *GI* has been already reported [52] and suggests that circadian rhythm is altered in response to blue light, turning on the Zeitlupe-mediated light signaling cascade, which results in the expression of hormone signaling and PAs biosynthesis. In addition, the upregulation of flavonoid genes like *4CL, UFGT* and peroxidases proposed as mitigators of short wavelengths, stability enhancers of the flavonoid mobilization as well as H_2_O_2_ detoxifiers, suggest that early stressing signals (linked to the expression of *ARRs*, flavonoid and peroxidase genes) allow a further increase of PAs production in cacao STPs [53, 54] (Fig. S7, Table S2).

Under long blue light exposure (d15-VS-d28) the PAs content depletion was correlated with the enrichment in high oxidative stress categories like phenylpropanoid biosynthesis, ascorbate and glutathione metabolisms and plant hormone signaling. Several peroxidases gene copies, flavonoid genes like *4CL*, and *INOSYTOL OXYGENASE* (*MIOX*) and *GLUTATHIONE-S TRANSFERASE* (*GST*) were upregulated at this time point. *MIOX* is involved in the biosynthesis of ascorbic acid to prevent ROS damage in plant cells [55]; meanwhile, *GST* is involved in vesicle trafficking-mediated transport of flavonoids [56], suggesting different mechanisms to cope with stress conditions. Furthermore, the *MYC2* TF and *JASMONOYL-L-AMINO ACID SYNTHETASE* (*JAR1*), associated with jasmonate signaling as well as *ARRs* genes like *ARR4*, *ARR9* and *ORR9* were significantly upregulated, supporting the occurrence of oxidative stress and the reduction in PAs content. Our observations support the idea that flavonoids undergo oxidation to overcome ROS-induced stress. The low PAs content after a long blue exposure triggered a second round of upregulation of most of the flavonoid structural genes. It is important to highlight that metabolite degradation is due to long blue light exposure alone and not light intensity, as the latter remains unchanged independently of the light treatment. In addition, the upregulation of *HY5* and *MYB12* under long blue light treatment suggests crosstalk between light and flavonoid regulation in large-scale systems [16, 57–59].

In STPs, the light and other physical variables (like the oxygen supply and agitation) cause hydrodynamic stress and likely added to the culture age will determine the final flavonoid yield in cacao cells. In our study, the interplay of different metabolic pathways together with the regulatory dynamic of flavonoid genes enabled to reach a total PAs content comparable to those levels reported for cacao seeds on day 15 [60]. In addition, the low PAs content at day 28 associated with a flavonoid reduction to overcome stress conditions allowed us to identify the critical point in which light generates an optimal cacao PAs production in STPs. The maintaining of PAs production until the end of the kinetics could be adjusted modifying conditions such as the intensity level or applying light pulses in short periods of time, in order to achieve a balance between the production of biomass and the concentration of metabolites of interest.

### Dynamics of light-driven ROS signals over flavonoids in STPs

Light-driven processes comprising both energy transfer and electron transport are accompanied by the formation of reactive oxygen species (ROS), including singlet oxygen (^1^O_2_), superoxide (O_2_^−^), hydrogen peroxide (H_2_O_2_) [44, 61]. Compared to superoxide and other ROS, H_2_O_2_ is the most stable molecule [62]. ROS are known to be metabolic byproducts in several organelles, including the mitochondria, peroxisome and chloroplast [63]. Our data suggest that in cacao cells grown in STPs the light-mediated ROS signals are initiated by superoxide anion type (O_2_^−^) followed by its fast conversion to H_2_O_2_ by *SOD* in the chloroplast, afterward, ROS signals can contribute to the dynamics of flavonoid production/degradation. Several results from the cacao transcriptome support such hypothesis. First chlorophyll/photosynthetic genes (*GluRS*, *GUN4, LHCA4*) were enriched at white and blue light exposure. *GluRS* and *GUN4* as well as of *F3H* and *DFR* are all regulated by *HY5*, suggesting a transcriptional regulatory hub for light and flavonoids [64–66]. Second, between the three ROS types evaluated, a larger proportion of markers associated with superoxide anion were upregulated throughout the experiment, concomitantly with the upregulation of *SOD* since treatment with white light. Third, the upregulation of *ANS* as a ROS marker in the superoxide ROS-type heatmap, suggests a stress response to light inputs. Furthermore, the high expression of *ANS* in cacao cells exposed to light treatments could be linked to PAs production. The data suggest that the conversion of O_2_^−^ into H_2_O_2_ occurred during the first days under white light and in the shift to blue light according to the *SOD* activity. In turn, the O_2_^−^-mediated ROS signals induced an increase of flavonoid production in vitro during the first two light treatments. Next, an excess of H_2_O_2_ accumulated at day 15 could promote the oxidation of flavonoids to overcome oxidative stress during d15-VS-d28, thus reducing the PAs content. At this point, the upregulation of *APX* and peroxidases suggest a support mechanism to the activity exerted by flavonoids in converting the excess of H_2_O_2_ into the non-toxic H_2_O molecule. Our data support an effect of light-mediated H_2_O_2_ induction quenched by flavonoid production in STPs, consistent with reports of increased secondary metabolite production in other cell culture systems [67, 68].

ROS markers were mainly downregulated in the course of the experiment, especially those downstream of the singlet oxygen and hydrogen peroxide. The reason for this is unclear, although Arabidopsis plants exposed to stress of growing in low and high-density on lighted chambers, showed similar results to our study, in terms of strong downregulation of defense genes and an upregulation of photosynthetic genes [69]. The authors suggested that both responses were a consequence of the reprogramming of metabolic activity to maximize cell growth under light conditions. Cacao cells in STPs are likely competing for nutrients, as well as light resources, resulting in the downregulation of ROS markers. Altogether, light-induced ROS type can be an indicator of the anion initiating the ROS signaling cascade in cell cultures, and it can provide specific ROS markers or ROS-related stress strategies that can be used to promote flavonoid production at large scale systems under light conditions.

### In silico light regulatory units (LRU) in promoters of cacao structural flavonoid genes

 The possibility of  light (non-mediated by transcription factors) directly regulating  late biosynthetic genes leading to the production of PAs in cacao cell cultures was explored. Direct light response of early biosynthetic genes (EBGs) *CHS*, *F3H*, *F3’H*, *DFR* could be explained by the presence of LRU in their promoter regions [18]. Here we show for the first time in silico that late biosynthetic genes (LBG) *ANS*, *ANR* and *LAR* also cointain LRU in their promoters, although with lower ratios of ACE/MRE components compared to EBGs. Multiple LRUs are considered to be redundant and additional elements act as enhancers of the light response [70]. In our data, *ANS_1* and *ANR_1,* the copies with higher expression levels in the transcriptome,  cointain two LRU, compared to the single LRU unit present in the *LAR* paralogs*.* Thus, the higher number of LRU in EBGs could provide a higher light-responsiveness to light stress conditions compared to LBGs. However, spacing requirements for the ACE/ACE or ACE/MYB core sequences as well as functional studies will be needed to assess the specific contributions of our findings to in vivo activation of LBG in response to light [71, 72].

### Crosstalk between light stress, ROS signaling and cacao flavonoids

In plants, catechins and PA polymers have been commonly associated with ROS scavengers against oxidative stress induced by herbivores and, most recently, by light [73, 74]. The crosstalk between light stress, ROS signaling and cacao flavonoid production has not been explored in detail, even though the light has a direct effect over cacao flavonoid production in cell cultures [9]. Our data of cacao cells growing in STPs show that PA production was positively correlated with the expression of *SOD* and negatively correlated with *APX* during the time course experiment, both genes encoding antioxidant enzymes. Also, gene expression levels of cacao flavonoid genes increased in response to light treatment with *CHS* and *ANS* showing the highest expression levels in vitro*.* We propose that light-induced ROS signals play crucial roles in activating flavonoid synthesis through an alternative non-enzymatic mechanism [75]. Simultaneously, enzymes like *SOD*, *APX*, catalases and peroxidases act together with flavonoids to detoxify H_2_O_2_ [75, 76]. Some studies have proposed the use of flavonoids as a mechanism of detoxification of hydrogen peroxide, instead of ascorbate, to convert H_2_O_2_ into water and molecular oxygen [77]. Our data point to plastids (like the chloroplast) as the first source of ROS species under light stress. Furthermore, the data suggest that flavonoids act as quenchers of H_2_O_2_, thus playing key roles in the cell homeostasis by keeping the concentration of H_2_O_2_ at a sub-lethal level [78]. These changes in organellar ROS levels can alter gene expression in the nucleus, as ROS can initiate retrograde signaling connecting light stress with transcriptional regulation [44].

### Model of the light effect of cacao cells producing flavonoids in STPs

**Fig. 7:**
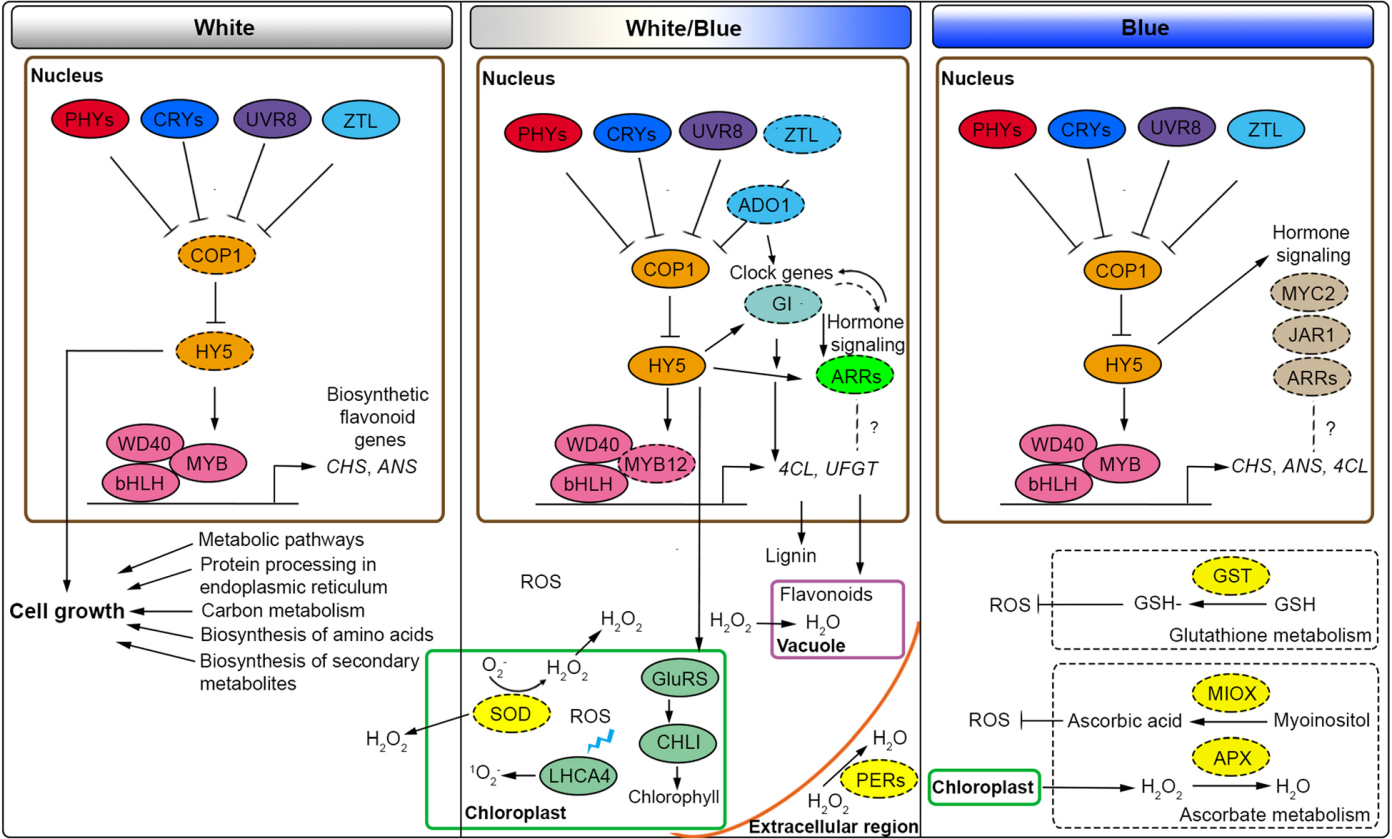
Proposed model of the flavonoid transcriptional regulation in cacao cells under light treatments in photobioreactors. Left panel: White light stimulates cell growth and the upregulation of flavonoid structural genes mediated by *COP1*/*HY5* light signaling pathway. Central panel: Shift to blue light, *COP1*/*HY5* continues to drive flavonoid biosynthesis. *MYB12* (as part of the MBW complex) mediates between light and flavonoids. *ADO1* (blue receptor), *GI* (circadian rhythm) and two-component regulators *ARRs* (hormone signaling) possibly interact to mediate flavonoid biosynthesis. Right panel: Long term exposure to blue light induces *COP1*/*HY5* pathway, as well as oxidative stress pathways ascorbate, glutathione, and two-component regulators. At the bottom, ROS production from chloroplast generates an excess of H_2_O_2_ stimulating flavonoid production early in the timecourse. H_2_O_2_ accumulation is detoxified by enzymatic (*SOD*, *APX*, *MIOX*, *PERs*) and non-enzymatic (flavonoids) mechanisms during and at the end of the experiment. Dotted ovals are candidate genes in the light network. *PHYs*, *CRYs*, *UVR8,* and *ZTL* are colored according to specific light qualities

Based on the available data on ROS gene markers, light signaling and flavonoid genes, we propose a model of transcriptional regulation of cacao PAs at bioreactor scale in response to the light treatments (Fig. 7). Under white light, cacao cell cultures are predominantly devoted to growth processes with most structural flavonoid genes being upregulated concomitantly with an increase of PAs content. Our data points that white light perception is mediated by *COP1*, coinciding with the upregulation of *SOD* activity in the chloroplast and the triggering of ROS signaling that stimulates the flavonoid biosynthesis. Next, the shift from white to blue light stimulated the expression of two-component regulators (*ARRs*) members of the circadian clock and hormone signals. These are early stress sensors and their expression coincides with a slight increase of PAs content. Long blue light exposure of cacao cells was a stress condition resulting in the degradation of PAs and the reactivation of a second flavonoid gene upregulation round to cope with harmful conditions. The upregulation of ascorbate and glutathione metabolic pathways suggested stressing conditions, where *APX* contributes in parallel with flavonoids to reduce the excess of H_2_O_2_ to H_2_O derived from *SOD* activity [79].

The transcriptomic data allow us to postulate candidate genes favoring the cacao PAs production at bioreactor scale such as *GluRS*, *GUN4*, *LHCA4* (chlorophyll biosynthesis), *GI* (circadian rhythm, ROS marker), *ARR4*, *ARR9*, *ORR9* of the two-component system (plant hormone signaling pathway) and *4CL*, *UFGT*, *CHS* and *ANS* (flavonoid pathway). These genes can be regulated directly or by complexes (like MBW formed by MYB, bHLH and WD40 TFs) downstream *HY5*, indicating that the *COP1* signaling pathway is mediating the light perception of cacao cells in STPs. In addition, the expression of ROS signaling markers during in vitro cultures indicates a positive effect of ROS molecules in the PAs biosynthesis at the beginning and the middle of the experiment, which turns negative at the end of the experiment when ROS stress becomes excessive. Understanding the effects of environmental factors such as light on the transcriptional network of flavonoids can improve the biotechnological approaches for secondary metabolite production of cell cultures at large-scale. The use of engineering techniques over candidate genes and/or balanced application of ROS- mediated stress could activate the flavonoid biosynthesis right until oxidation starts to occur. Both strategies are proposed as alternative ways of target metabolites production independent of the variations imposed by climatic conditions *in planta*.

## Conclusions

This study is the first report on transcriptomic profiles of cacao cell cultures under light treatments at bioreactor level. In this study PAs production was positively correlated with *ADO1*, a blue photoreceptor supporting the *COP1-*driven light regulation model mechanism. In addition, light-mediated ROS signals were initiated by superoxide anion followed by its fast conversion into the stable molecule H_2_O_2_ by *SOD* in the chloroplast. The accumulation of ROS potentially controls the dynamic of flavonoid production/degradation. This result suggests that flavonoids could act like quenchers of H_2_O_2_, performing a function against oxidative stress induced by light in cacao cell cultures. The presented model includes the crosstalk between light-regulated genes, ROS markers and flavonoids where light signaling *COP1*-mediated is the ruling mechanism under our experimental conditions. Taken together, this study allowed us to enhance the comprehension of the gene expression of cacao cells exposed to light abiotic stress. The results will further facilitate the design of biotechnological strategies to boost and modulate the in vitro flavonoid production at large-scales.

## Methods

### Plant material and growth conditions

Cacao cell cultures were established from Trinitario ecotype (coded as BIOA) collected in the commercial crops of Compañía Nacional de Chocolates, San Vicente de Chucurí - Santander, Colombia (06° 53′ 00“N; 73° 24’ 50” W). Cell cultures were established following Rojas et al. [68]. A cacao cell line (CB-1) derived from friable cotyledonar calli was used to initiate the suspensions. Two hundred ml of liquid media were inoculated with 16 g of calli (fresh weight) in a 1 L flask. Cultures were maintained for 28 days with a subculture at day 14. The cultures were maintained at 23 ± 2 °C under cool-white fluorescent lamps (12 μmol m^− 2^ s ^− 1^) for 16 h light at 100 rpm. Three hundred ml of pelleted cells from the flask cultures were used to inoculate each of three 7.5-L stirred tank bioreactors (New Brunswick™ BioFlo®/ CelliGen® 115) with 5 L work volume, adapted with a pitched blade propeller and a micro-bubble sparger at 1.0 LPM. The conditions were set to 100 rpm and 22 ± 1 °C.

### Light treatments

Bioreactors were adapted with a customized home-made light jacket (Bioin Soluciones S.A.S) to provide the light conditions in a fed-batch process. Cacao cells in STPs were subjected to a sequential exposure of white LED (W) (459-700 nm wavelength spectrum, 60 μmol/m^2^/s) followed by exposure to blue LED (B) (450-455 nm wavelength spectrum, 60 μmol/m^2^/s) 14 days each. A subculture step was performed in the shift from white to blue light to feed the bioreactor with fresh media. Three light treatments were defined as white light (d0-VS-d14) during 14 days, followed by a shift to blue light (d14-VS-d15) and 15 days of blue light (d15-VS-d28). White and blue LED arrays were designed to give the set intensity, under a photoperiod of 16:8 light to dark. The monitoring and control of the batch cultures were carried out using the BioCommand® software. Bioreactors were sampled at days 0, 14, 15 and 28 during the time course. Three replicates were collected for each time point from three independent bioreactor experiments. Samples were preserved in RNA Later® (ratio 1:5) and stored at − 20 °C.

### Quantification of total proanthocyanidins (PAs)

Extraction and quantification of total proanthocyanidins were performed according to Liu et al. [60]. Soluble and insoluble PAs were extracted sequentially from the same samples consisting of 50 mg of dry weight cacao cell suspensions previously lyophilized at − 20 °C with 1.5 ml of extraction solution (70% acetone: 29.5% water: 0.5% acetic acid). To quantify soluble PA levels, the p-dimethylamino-cinnamaldehyde (DMACA) method was used [80]. For insoluble PAs quantification, cyanidin equivalents were calculated using a cyaniding chloride calibration curve following Liu et al. [60]. Total PAs content was determined as the sum of both measurements. Three biological replicates from each light treatment were extracted. Statistical analysis of the proanthocyanidin content was evaluated using *rethinking R package* and 95% probability intervals to obtain significant comparisons among treatments.

### RNA extraction, library preparation and sequencing

Three biological samples, one for each bioreactor, were sampled at each time point (d0, d14, d15, d28) for a total of 12 samples. Total RNA was extracted from each sample taking 0.1 ml approximately of pellet volume using combined protocols between Pure Link Plant RNA Reagent (Ref: 12322012, Thermo Fisher Scientific) and RNeasy Plus Universal Mini Kit (Cat No. 73404, QIAGEN). RNA concentration and quality parameters were measured using an Agilent 2100 bioanalyzer. The construction of the cDNA libraries and RNAseq was performed by the Genomics Core Facility at Penn State University (University Park, USA). cDNA libraries were prepared with a TruSeq stranded mRNA library prep Kit (cat# RS-122-2101, Illumina, San Diego, CA, USA). The libraries were sequenced on a HiSeq™ 2500 (Illumina) using single-end runs of 100 nt.

### Mapping and functional annotation

Quality parameters including GC-content and phred scores were applied to filter the reads. After raw reads were filtered to exclude those with low complexity, mapping to the Criollo Cacao Genome V2 (CIRAD, 2017) was performed by using HISAT [81]. The sequences were annotated using Blast2Go (e-value < 10^− 5^) and the Cacao genome as reference [82]. The following public protein databases were used for the functional classification: Clusters of Orthologous Groups (COGs) [83], Kyoto Encyclopedia of Genes and Genomes (KEGG) [84], and the Gene Ontology (GO) protein database [85]. Gene expression levels were normalized and differential expression analyses were conducted using Deseq2. Comparison between days in culture was established as a condition in the differential expression model (dds = DESeqDataSetFromMatrix(countData = countData, colData = colData, design = ~condition), the [86, 87]. Three pairwise comparisons were performed to identify differentially expressed genes (DEGs) over the time course of the experiment. Comparisons were made between day 0 and 14 (d0-VS-d14), day 14 and 15 (d14-VS-d15) and day 15 and 28 (d15-VS-d28). Statistical significance of gene expression between pairwise comparisons was determined to analyze integral read counts per gene with DESeq2. DEGs were identified as those having a *p*-value < 0.05. These putative DEGs were subjected to Gene Ontology (GO) enrichment analysis and KEGG Pathway enrichment analysis to investigate functions and pathways affected over the time course. GO and KEGG enrichment analyses were performed using KOBAS software [88] to test the statistical enrichment of terms associated with DEGs. An FDR < 0.05 was used as the threshold to determine significant GO/KEGG enrichment of the gene sets. Additionally, we used the Plant Transcription Factor Database [89] to annotate DEGs with reference to Arabidopsis. Finally, a cluster analysis of expression patterns for DEGs using the Short Time-series Expression Miner (STEM) software was performed to understand the changes in absolute expression during light exposure of cell cultures in the bioreactors [90].

### In silico analysis of flavonoid structural gene promoters

To identify putative direct light regulation on the cacao flavonoid pathway genes, we searched for LRUs in the promoters of the structural genes in the pathway. For this, two orthologs were chosen based on their contrasting expression levels in the transcriptome. Thus, the promoter sequences of chalcone synthase *CHS* (*CHS_1* Tc04v2_g017080, *CHS_2* Tc04V2_g004230); chalcone isomerase *CHI* (*CHI_1* Tc10v2_g004990, *CHI_2* Tc01V2_g001600); flavanone 3 hydroxylase *F3H* (Tc01v2_g001580); flavonoid 3′-hydroxylase *F3’H* (*F3’H_1* Tc03v2_g08270, *F3’H _2* Tc09V2_g006700); flavanol synthase *FLS* (*FLS_1* Tc05v2_g015820, *FLS_2* Tc08v2_g010180); dihydroflavonol 4-reductase *DFR* (*DFR_1* Tc08v2_g002430, *DFR_2* Tc04V2_g011490); anthocyanidin synthase *ANS* (*ANS_1* Tc03v2_g022320, *ANS_2* Tc05V2_g001290); anthocyanidin reductase *ANR* (Tc06v2_g017140) and leucoanthocyanidin reductase *LAR* (*LAR_1* Tc03v2_g002030, *LAR_2* Tc02V2_g030980) were retrieved from the NCBI database for *Theobroma cacao* genome V2. Genomic regions up to 3000 bp upstream of the start codon of each gene were selected of genomic regions using the genome data viewer from GenBank (http://www.ncbi.nlm.nih.gov/Genbank/) and then exported as nucleotide fasta files to be processed using Geneious software [91]. We searched for any ACE or MRE elements in each promoter sequence of the cacao flavonoid genes. This was done by blasting the consensus sequences of the Arabidopsis model CACGT (ACE) and ACCTACC (MRE) from Arabidopsis in each cacao promoter sequence using the Genious software. Furthermore, a gene map was generated representing each flavonoid gene structure here selected.

### Photoreceptors and light signaling elements

In the cacao transcriptome, we analyzed photoreceptors genes encoding phytochromes (PHYs), cryptochromes (CRYs), phototropins (PHOTs), UV receptor and Zeitlupes (ZTL/FKF1/LPK2 or Adagio proteins, ADO) as well as downstream light-responsive elements to identify key genes of the light transduction mediated by the *COP1* signaling cascade in the cacao flavonoid production. We determined the levels of expression for six red/far red responsive genes (*PHYA*, *PHYB1*, *PHYB2*, *PHYC*, *PHYD,* and *PHYE*), eight blue/UV responsive genes (*CRY1*, *CRY2*, *CRY3*, *PHOT1*, *PHOT2*, *UV Resistance 8 UVR8*, *ADO1*, *ADO3*) and nine downstream light signaling genes like *COP1*, *COP1.1, COP10*, suppressor of *PHYA* (*SPA1*, *SPA2*), *HY5*, gibberellic acid-insensitive *GAI1* (*DELLA*), early flowering *3 ELF3* and *MYB12* along with the time course experiment.

### Reactive species oxygen (ROS) markers

To identify ROS markers involved in light signaling in the cell cultures, cacao DEGs were compared to 832 abiotic stress ROS gene markers previously reported for Arabidopsis. The Arabidopsis ROS markers coding sequences were retrieved from TAIR database (https://www.arabidopsis.org/). Hits were searched independently for each type of ROS, with cacao DEGs blast searches against 323 singlet oxygen (^1^O_2_), 205 superoxide anion (O_2_^−^) and 304 hydrogen peroxide (H_2_O_2_) Arabidopsis gene markers. Additionally, we identified within the cacao ROS markers, those genes codifying for transcription factors (TFs) to carry out specific analyzes. A KEGG metabolic pathway analysis of the resulting cacao ROS markers was further performed using KOBAS software. The data were represented on a heatmap using R software.

### Quantitative PCR

Real-time quantitative reverse transcription-PCR (qRT-PCR) was used to validate gene expression patterns identified by the RNA-Seq analysis. Aliquots from the RNA were treated with RNase-free DNase (Promega, Cat. M6101) to remove potential genomic DNA contamination following the manufacturer’s protocol. A total of 3 μg of treated RNA were reverse transcribed by MMuLV Reverse Transcriptase (New England Biolabs, Ipswich, MA, USA) using oligo-(dT)15 primers. Specific amplification was done for *CHS*, *DFR*, *ANS*, *ANR*, *LAR* and *NAC* and primers were designed based on the sequences available for the Criollo Cacao genome database. *GAPDH* was selected as the endogenous reference. qRT-PCR was performed in a total reaction volume of 10 μL containing 4 μL of diluted cDNA (1:8), 5 μL of SYBR Premix, 0.2 μL of Rox (TaKaRa, Mountain View, CA, USA), and 0.4 μL of each 5 μM primer. Four technical replicates were made for each sample. qRT-PCR was conducted in a real-time PCR System (Applied Biosystem Step One Plus, Nutley, NJ, USA) with the following parameters: 10 min at 95 °C, followed by 40 cycles of 15 s at 95 °C and 60 s at 60 °C followed by a melting curve of 15 s at 95 °C, 60 s at 60 °C and 15 s at 95 °C with a 1 °C ramp rate.

## Supplementary Information

**Additional file 1: Figure S1.** Histogram of gene ontology classification for all mapped genes. **Figure S2.** Histogram of genes COG classification for all mapped genes. **Figure S3.** Overview of DEGs in three pairwise comparisons for cacao cell bioreactors under light treatments. **Figure S4.** Cluster analysis of DEGs in cacao cell bioreactor under light treatments and significant KEGG enrichment listed for each cluster (*p*-value <0.05). * Category enriched for cluster 39 has *p*-value < 0.1. **Figure S5.** Clusters obtained for DEGs of cacao cell bioreactor under light treatments using STEM. Thirty-five clusters were generated. Significant clusters (*p* <0.05) are highlighted in colors. **Figure S6.** Expression patterns of antioxidant enzymes of cacao cells in STPs. SOD: Superoxide dismutase, CAT: Catalase, APX: Ascorbate peroxidase. **Figure S7.** Expression patterns of flavonoid biosynthetic genes identified in cacao cells in STPs. Different locus for a same gene are colored equal. **Figure S8.** qRT-PCR analysis of differentially expressed genes in cacao cell suspensions. Transcript levels and qRT-PCR results of 8 randomly selected genes from RNA-sequencing. The left y-axis shows the relative gene expression levels analyzed by qPCR (gray columns). The right y-axis indicates the corresponding expression data of RNA-seq (black dots). The x-axis represents the time (days) of light/dark exposure. Bars represent SD (*n* = 3).

**Additional file 2: Table S1.** Mapping of *Theobroma cacao* RNA-seq library reads to the Cacao criollo reference genome database. **Table S2.** KEGG enrichment annotation for the three pairwaise comparisons in White and Blue conditions. **Table S3.** List of Transcription factors identified in the DEGs of cacao cells in light-treated Bioreactors. **Table S4.** Annotation and statistical information of photoreceptors identified in the cacao cell bioreactors. **Table S5.** KEGG metabolic enrichment analysis for ROS markers in cacao DEGs. **Table S6.** Up and down regulated anotated ROS markers in cacao DEGs for the three pairwise comparisons. Red shadow locus: Upregulated. Blue shadow locus: Downregulated. **Table S7.** Cacao transcription factor identified in each of three ROS types. **Table S8.** Primers used for qRT-PCR analysis of flavonoid biosynthesis related genes

## Data Availability

All the sequencing data generated in this study were submitted to NCBI SRA Submission-ID: SUB8417929, with BioProject ID: PRJNA742476 and BioSample accession: SAMN19955633.

## Abbreviations

DEGs: Differentially expressed genes
PAs: Proanthocyanidins
ROS: Reactive oxygen species
STP: Stirred tank bioreactor
