# Supplementary material for: Transcriptomic analyses of cacao flavonoids produced in photobioreactors
Source: BMC Genomics. 2021 Jul 19;22:551. doi: 10.1186/s12864-021-07871-0 (PMC8287782; doi:10.1186/s12864-021-07871-0)
Supplement: Supplementary file 1 — Additional file 1: Figure S1. Histogram of gene ontology classification for all mapped genes. Figure S2. Histogram of genes COG classification for all mapped genes. Figure S3. Overview of DEGs in three pairwise comparisons for cacao cell bioreactors under light treatments. Figure S4. Cluster analysis of DEGs in cacao cell bioreactor under light treatments and significant KEGG enrichment listed for each cluster (p-value <0.05). * Category enriched for cluster 39 has p-value < 0.1. Figure S5. Clusters obtained for DEGs of cacao cell bioreactor under light treatments using STEM. Thirty-five clusters were generated. Significant clusters (p <0.05) are highlighted in colors. Figure S6. Expression patterns of antioxidant enzymes of cacao cells in STPs. SOD: Superoxide dismutase, CAT: Catalase, APX: Ascorbate peroxidase. Figure S7. Expression patterns of flavonoid biosynthetic genes identified in cacao cells in STPs. Different locus for a same gene are colored equal. Figure S8. qRT-PCR analysis of differentially expressed genes in cacao cell suspensions. Transcript levels and qRT-PCR results of 8 randomly selected genes from RNA-sequencing. The left y-axis shows the relative gene expression levels analyzed by qPCR (gray columns). The right y-axis indicates the corresponding expression data of RNA-seq (black dots). The x-axis represents the time (days) of light/dark exposure. Bars represent SD (n = 3). [file 12864_2021_7871_MOESM1_ESM.docx]

Transcriptomic Analyses of Cacao Flavonoids Produced in Photobioreactors

**Adriana M. Gallego^1^, Luisa F. Rojas^2^, Wilmar G. Valencia^3^, Lucía Atehortúa^1^, Aura I. Urrea^1^, Andrew S. Fister^4^, Mark J. Guiltinan^4^, Siela N. Maximova^4^, and Natalia Pabón-Mora^5^***

1. Grupo de Biotecnología, Instituto de Biología, Universidad de Antioquia, Medellín, Colombia

2. Grupo de Biotransformación, Escuela de Microbiología, Universidad de Antioquia, Medellín, Colombia

3. Centro de Investigación, Desarrollo y Calidad CIDCA. Compañía Nacional de Chocolates S.A.S. Km 2 Vía Belén autopista Medellín-Bogotá.

4. Department of Plant Science, Pennsylvania State University, University Park, PA, USA; Huck Institutes of the Life Sciences, Pennsylvania State University, University Park, PA, USA

5. Grupo Evo-Devo en Plantas, Instituto de Biología, Universidad de Antioquia, Medellín, Colombia


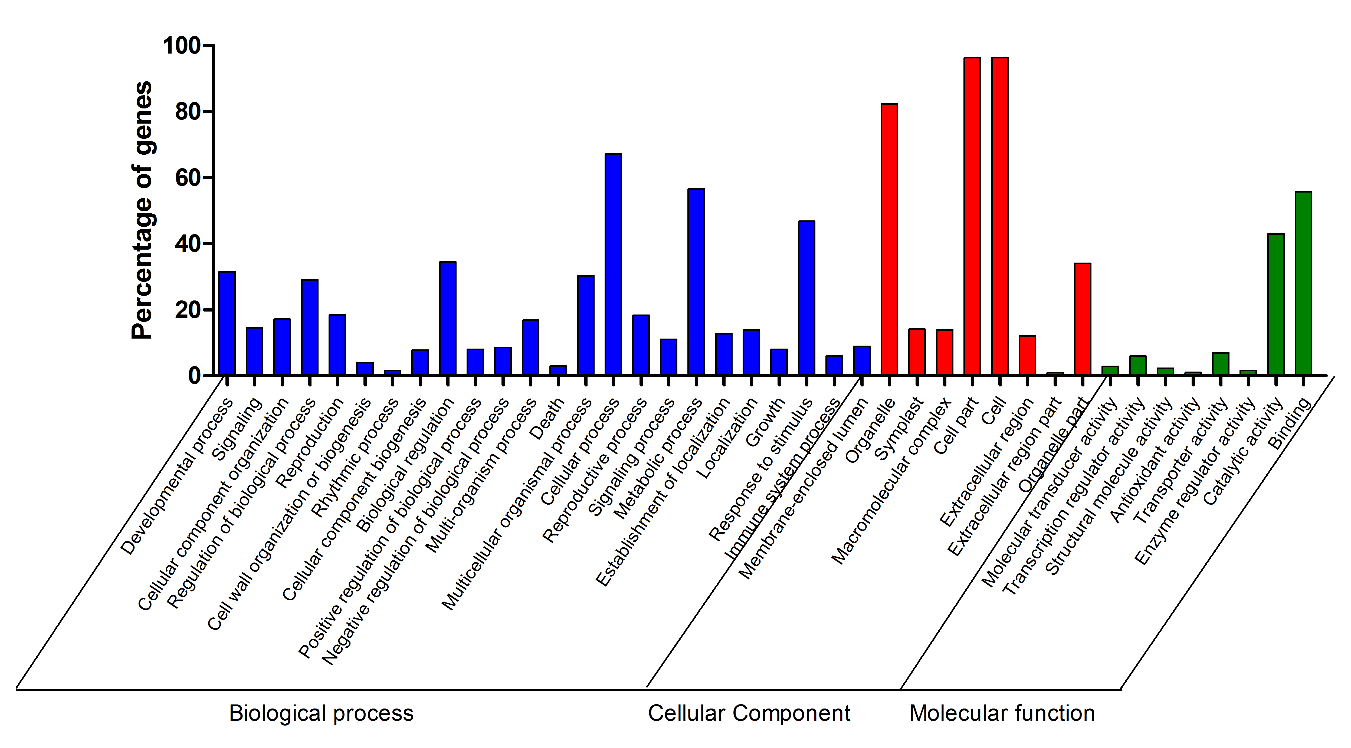


**Figure S1.** Histogram of gene ontology classification for all mapped genes


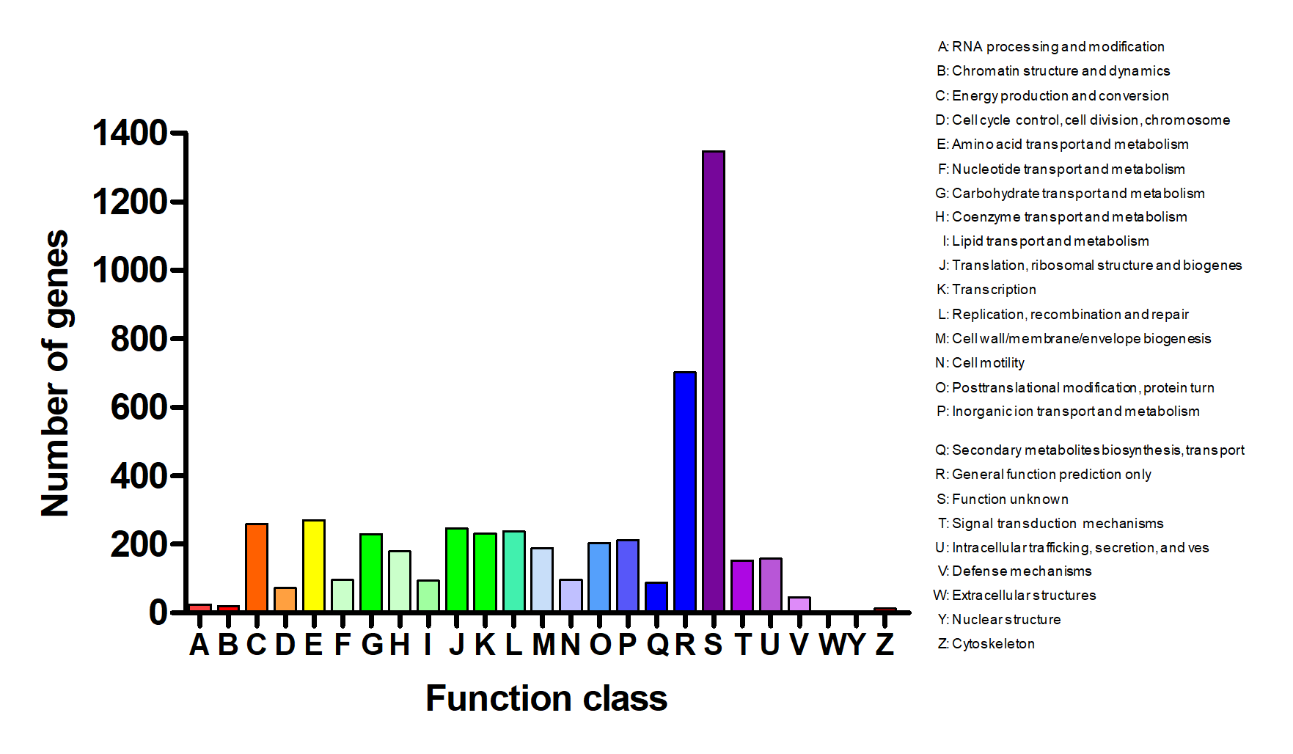


**Figure S2.** Histogram of genes COG classification for all mapped genes


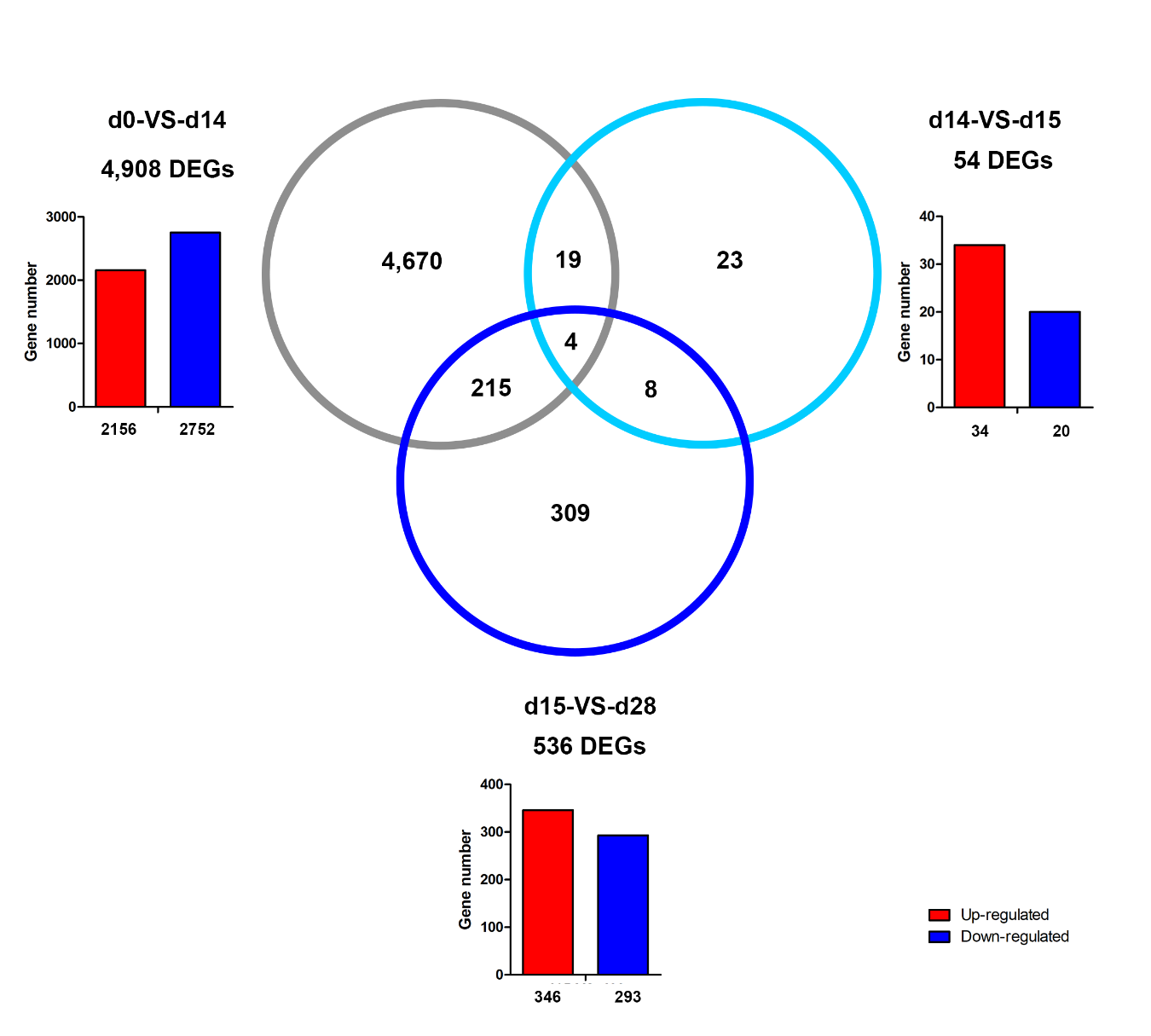


**Figure S3.** Overview of DEGs in three pairwise comparisons for cacao cell bioreactors under light treatments.


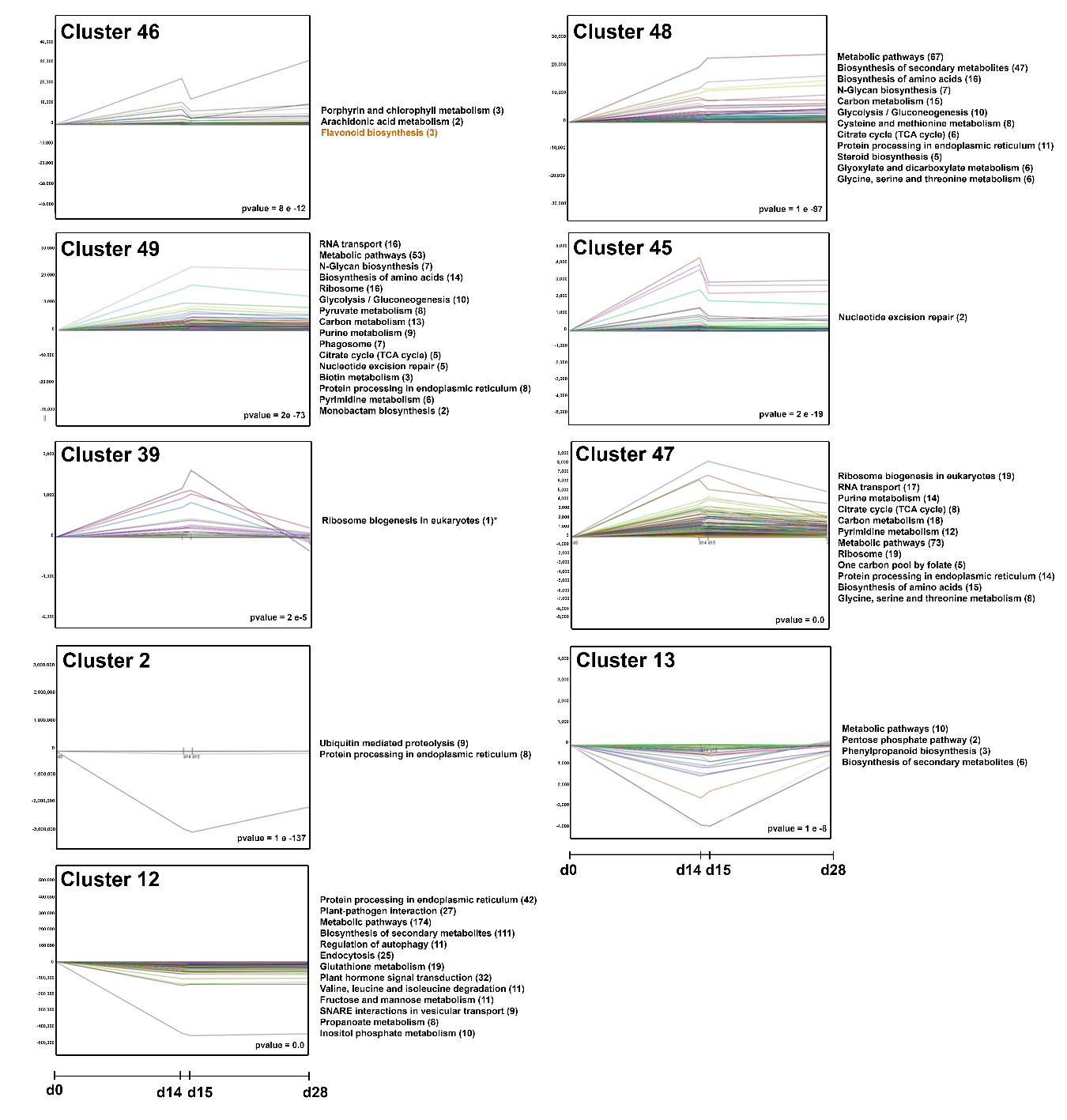


**Figure S4.** Cluster analysis of DEGs in cacao cell bioreactor under light treatments and significant KEGG enrichment listed for each cluster (p-value <0.05). * Category enriched for cluster 39 has p-value < 0.1.


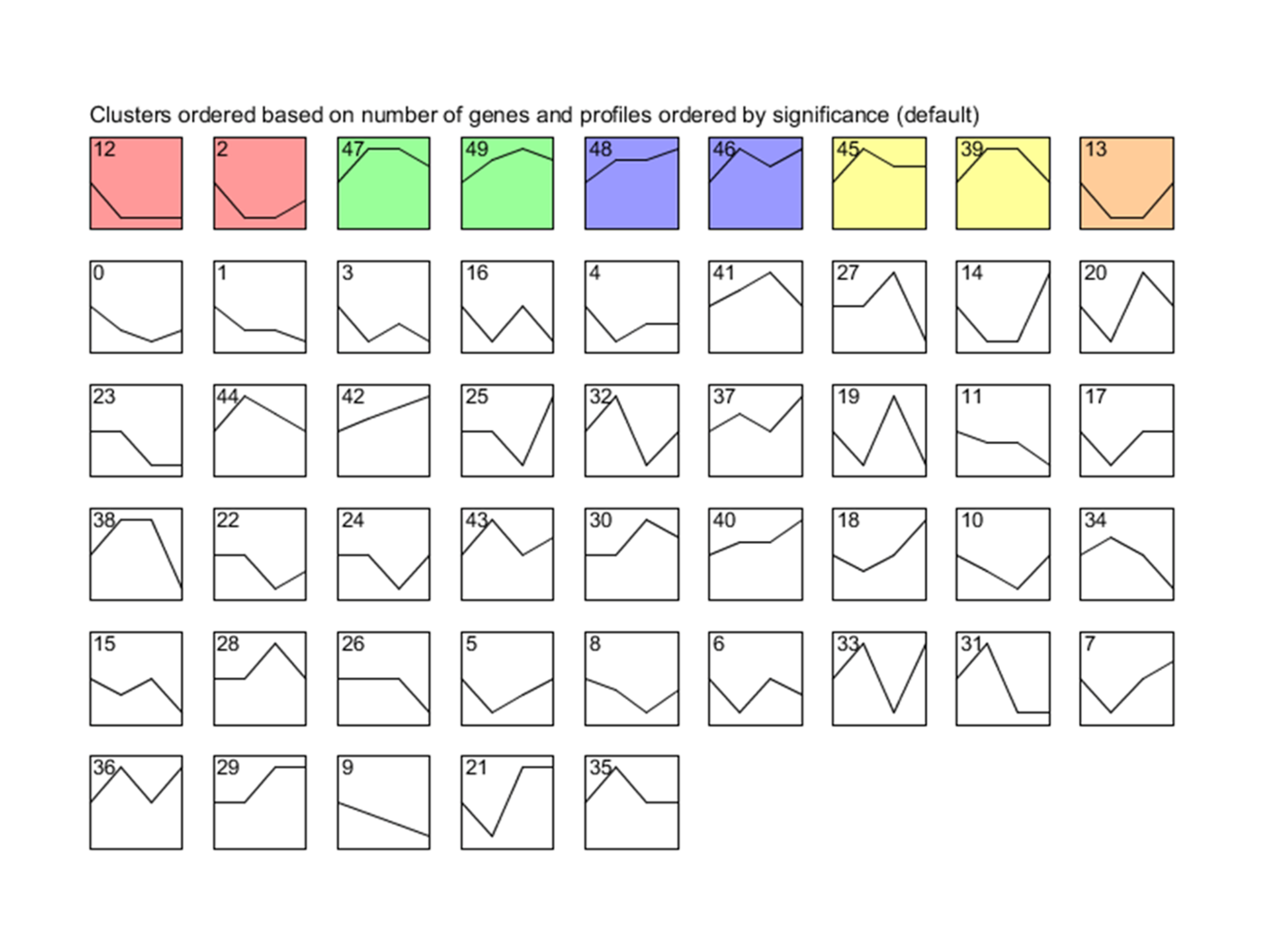


**Figure S5.** Clusters obtained for DEGs of cacao cell bioreactor under light treatments using STEM. Thirty-five clusters were generated. Significant clusters (p <0.05) are highlighted in colors.


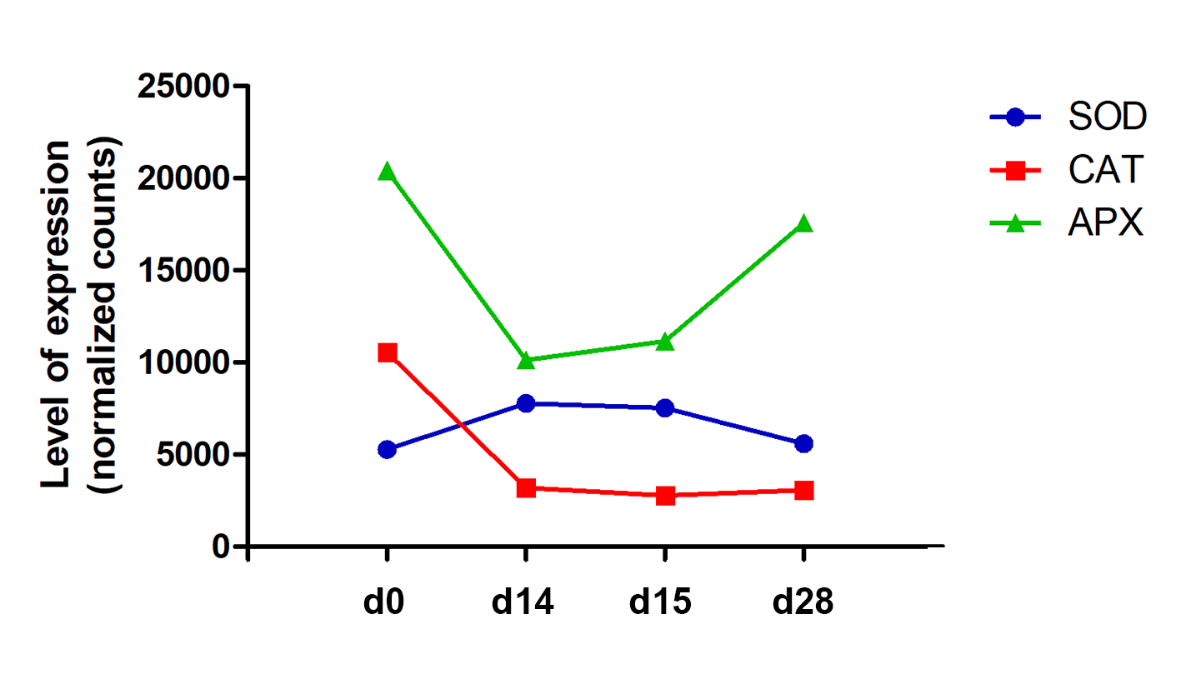


**Figure S6.** Expression patterns of antioxidant enzymes of cacao cells in STPs. SOD: Superoxide dismutase, CAT: Catalase, APX: Ascorbate peroxidase.


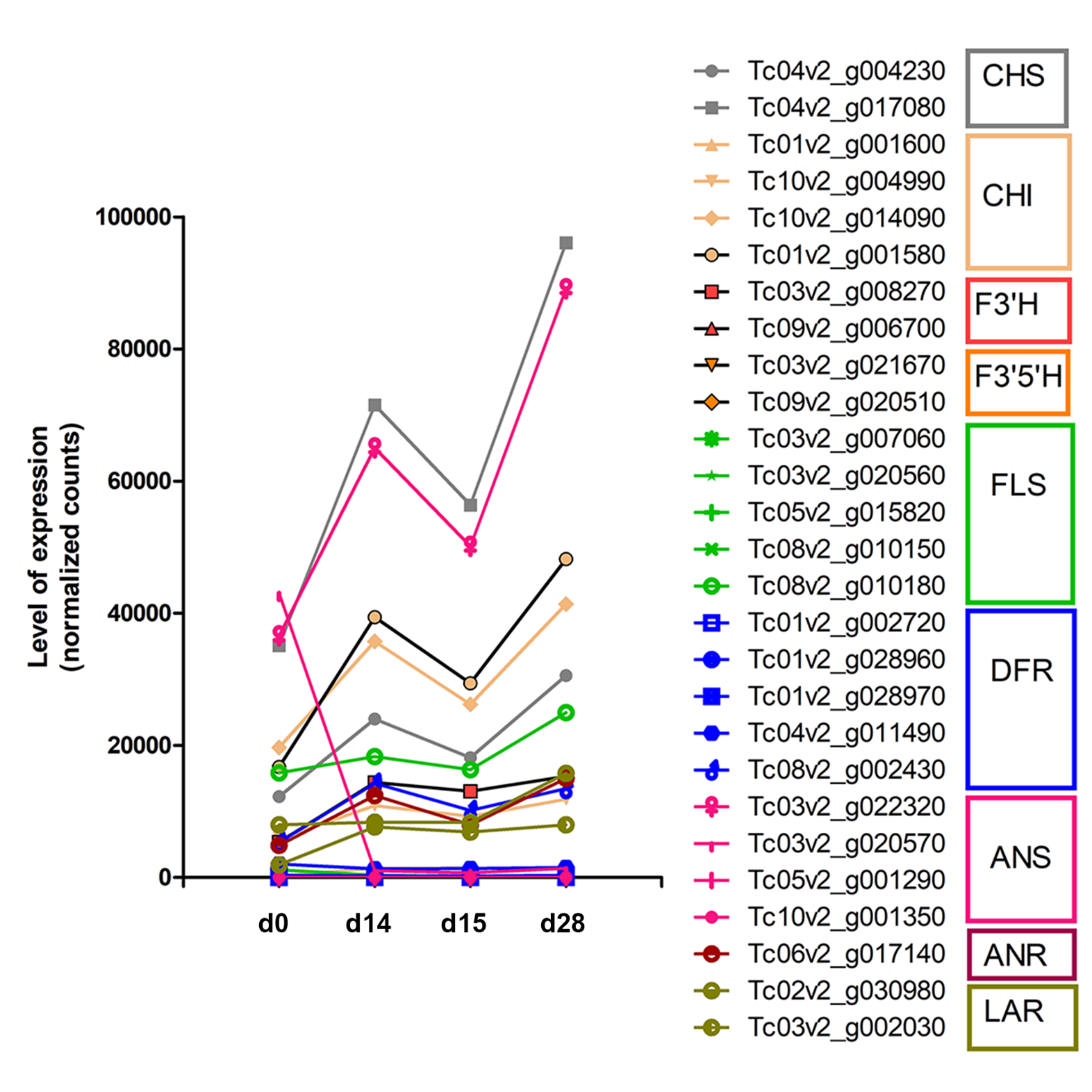


**Figure S7.** Expression patterns of flavonoid biosynthetic genes identified in cacao cells in STPs. Different locus for a same gene are colored equal.


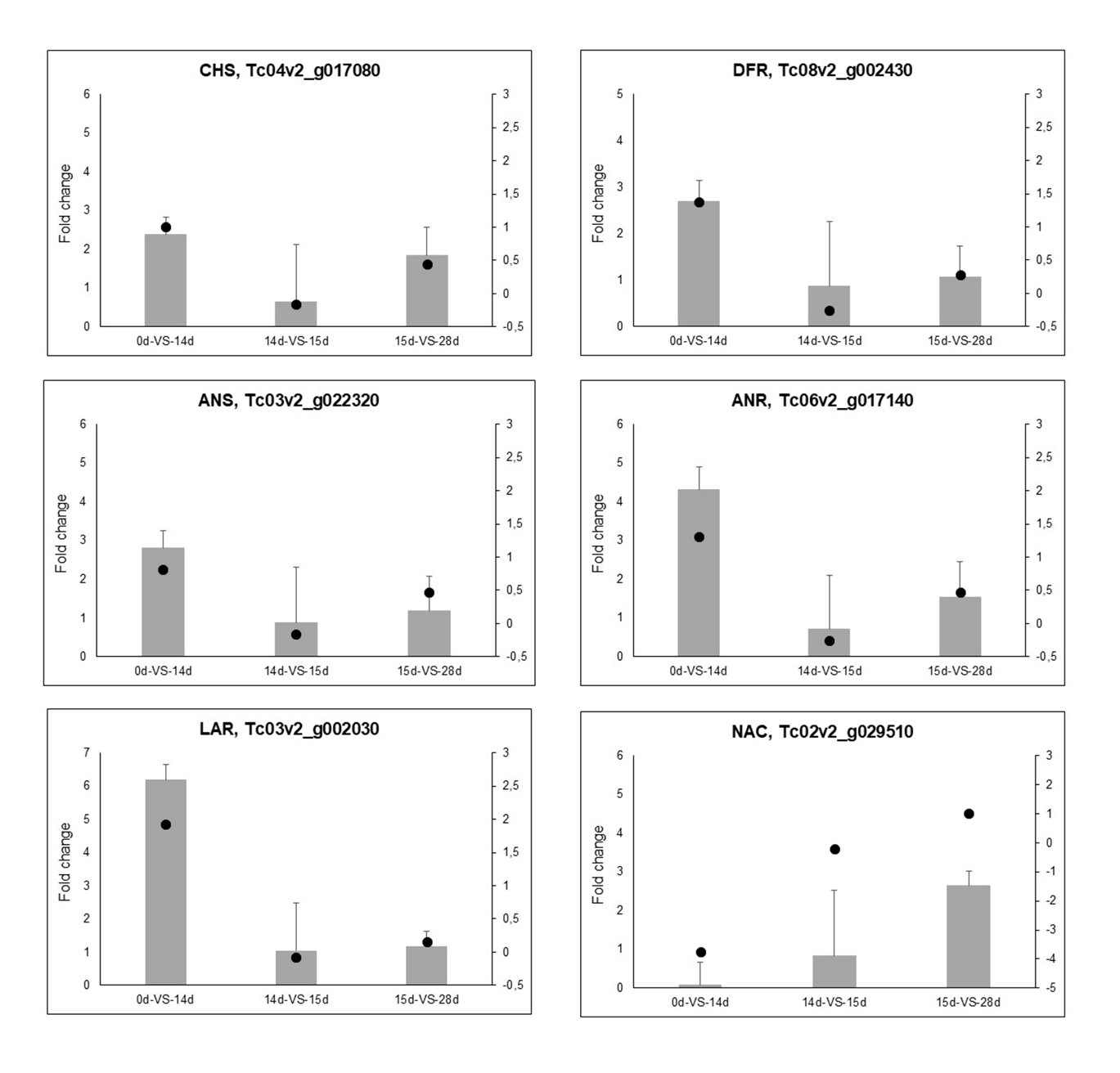


**Figure S8. qRT-PCR analysis of differentially expressed genes in cacao cell suspensions.** Transcript levels and qRT-PCR results of 8 randomly selected genes from RNA-sequencing. The left y-axis shows the relative gene expression levels analyzed by qPCR (gray columns). The right y-axis indicates the corresponding expression data of RNA-seq (black dots). The x-axis represents the time (days) of light/dark exposure. Bars represent SD (n = 3).
